# NF-κB Activation in Lymphoid Malignancies: Genetics, Signaling, and Targeted Therapy

**DOI:** 10.3390/biomedicines6020038

**Published:** 2018-03-26

**Authors:** Paula Grondona, Philip Bucher, Klaus Schulze-Osthoff, Stephan Hailfinger, Anja Schmitt

**Affiliations:** Interfaculty Institute for Biochemistry, Eberhard Karls University of Tuebingen, Hoppe-Seyler-Str. 4, 72076 Tuebingen, Germany; paula.grondona@student.uni-tuebingen.de (P.G.); philip.bucher@student.uni-tuebingen.de (P.B.); KSO@uni-tuebingen.de (K.S.-O.); anja.schmitt@ifib.uni-tuebingen.de (A.S.)

**Keywords:** NF-κB, lymphoma, leukemia, CARMA1, CARD11, CD79, MyD88

## Abstract

The NF-κB transcription factor family plays a crucial role in lymphocyte proliferation and survival. Consequently, aberrant NF-κB activation has been described in a variety of lymphoid malignancies, including diffuse large B-cell lymphoma, Hodgkin lymphoma, and adult T-cell leukemia. Several factors, such as persistent infections (e.g., with *Helicobacter pylori*), the pro-inflammatory microenvironment of the cancer, self-reactive immune receptors as well as genetic lesions altering the function of key signaling effectors, contribute to constitutive NF-κB activity in these malignancies. In this review, we will discuss the molecular consequences of recurrent genetic lesions affecting key regulators of NF-κB signaling. We will particularly focus on the oncogenic mechanisms by which these alterations drive deregulated NF-κB activity and thus promote the growth and survival of the malignant cells. As the concept of a targeted therapy based on the mutational status of the malignancy has been supported by several recent preclinical and clinical studies, further insight in the function of NF-κB modulators and in the molecular mechanisms governing aberrant NF-κB activation observed in lymphoid malignancies might lead to the development of additional treatment strategies and thus improve lymphoma therapy.

## 1. NF-κB in Lymphocytes

The NF-κB transcription factors are involved in the regulation of a variety of biological processes, such as inflammation, survival, and proliferation. The NF-κB family comprises five structurally related members forming different homo- or heterodimers: RelA (also known as p65), c-Rel, RelB, NF-κB1 (p50 and its precursor p105), as well as NF-κB2 (p52 and its precursor p100). The NF-κB proteins share a conserved REL homology domain required for homo- or heterodimerization, the interaction with inhibitor of κB (IκB) proteins, nuclear localization, and DNA binding. In quiescent cells, the inactive transcription factors are retained in the cytoplasm either by binding to the classical IκB proteins IκBα, IκBβ, and IκBε or by interaction with the inactive precursors p105 and p100. NF-κB activation in response to extracellular cues is regulated by two distinct pathways: In the canonical NF-κB pathway, stimulus-dependent activation of the IκB kinase (IKK) complex, comprising the catalytic subunits IKKα and IKKβ as well as the regulatory subunit IKKγ (also known as NF-κB essential modulator; NEMO), results in the phosphorylation and subsequent proteasomal degradation of the IκB proteins [1,2]. This allows the nuclear translocation of the NF-κB transcription factors, preferentially heterodimers of p50 and RelA or c-Rel, as well as their subsequent DNA binding and target gene transcription. In normal lymphocytes, stimulation-induced NF-κB activation is only transient and rapidly terminated by feedback inhibition involving the NF-κB-dependent expression of negative regulators, such as IκBα, IκBε, and A20 [3]. In contrast, activation of the non-canonical pathway involves the NF-κB inducing kinase (NIK)-dependent activation of IKKα and the inducible proteolytic processing of the precursor protein p100 to generate p52, which preferentially forms transcriptionally active heterodimers with RelB [2,4]. In addition to the classical IκB proteins, the IκB family also includes the atypical or nuclear IκB proteins BCL3, IκBζ, IκBNS, and IκBη, which are normally expressed only in response to pro-inflammatory stimuli. Unlike the classical IκBs that function as cytoplasmic inhibitors, the atypical IκB proteins primarily act as transcriptional co-activators or co-repressors in the nucleus, where they modulate the expression of a subset of NF-κB target genes [5,6,7].

In lymphocytes, NF-κB signaling is transiently activated in response to engagement of various receptors, such as antigen receptors, TNF receptors as well as interleukin-1 (IL-1) and Toll-like receptors (TLRs), and plays a critical role in development, survival, and acquisition of effector functions [8]. A variety of lymphoid malignancies, however, exhibits pathological activation of NF-κB due to diverse genetic lesions which affect key components of the NF-κB signaling pathway [9,10]. The first evidence linking core components of the NF-κB signaling pathway to lymphomagenesis has been reported in studies on the viral oncogene product v-Rel which causes aggressive lymphomas in birds and other animals [11,12]. Several subsequent studies have identified genetic aberrations in the NF-κB protein family, such as amplifications in the *REL* locus, in different lymphoid cancers [13,14]. To date, several lymphoid malignancies, such as Hodgkin lymphoma (HL), diffuse large B-cell lymphoma (DLBCL) of the activated B cell-like (ABC) subtype, lymphomas of the mucosa-associated lymphoid tissue (MALT), primary mediastinal B-cell lymphoma (PMBL), mantle cell lymphoma (MCL), multiple myeloma, and chronic lymphocytic leukemia (CLL), have been associated with aberrant NF-κB signaling [15,16,17,18,19,20,21,22,23,24]. Whereas genetic aberrations affecting the NF-κB members themselves are relatively rare, deregulated NF-κB activation is frequently achieved by oncogenic events which trigger the constitutive activity of various upstream signaling pathways, culminating in enhanced transcriptional activity of NF-κB [9,25]. In this review, we will describe recurrent genetic lesions driving pathological NF-κB activation in lymphoid malignancies. We will particularly focus on the molecular mechanism of the affected, aberrantly expressed regulators as well as their impact on the composition and function of the signaling complexes involved in NF-κB regulation. The exact molecular characterization of the key oncogenic mechanisms of constitutive NF-κB activation either shared by several or unique to certain lymphoid malignancies might allow the rational design of therapeutic strategies tailored to the specific tumor entities and might thus significantly improve lymphoma therapy.

## 2. Oncogenic MyD88 Mutations

**Figure 1:**
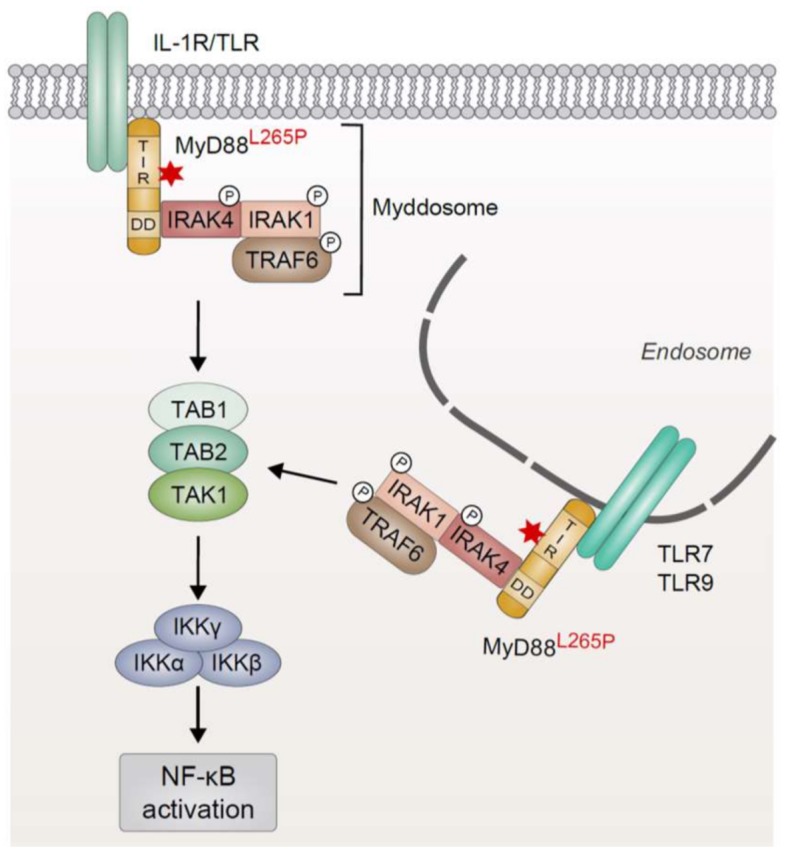
Oncogenic MyD88 mutations activate the canonical NF-κB pathway. Activation of canonical NF-κB signaling can be induced by the triggering of members of the IL-1 receptor or the Toll-like receptor family, which localize to the cell surface or, in the case of TLR7 and TLR9, to the endosomal compartment. Ligand binding induces the recruitment of MyD88 to the activated receptor via its TIR domain and triggers subsequent downstream signaling. MyD88 oligomers nucleate the formation of the Myddosome which ultimately results in the activation of IRAKs and the recruitment of the E3 ubiquitin ligase TRAF6. TRAF6 in turn recruits and activates TAK1 which mediates activation of the IKK complex resulting in canonical NF-κB activity. The oncogenic variant MyD88^L265P^ promotes spontaneous oligomerization and activation of downstream signaling independently of receptor stimulation. Mutant MyD88 is denoted with a red asterisk. DD, death domain; IL-1R, interleukin-1 receptor; IKK, IκB kinase; IRAK, IL-1R-associated kinase; MyD88, myeloid differentiation primary response protein 88; TAK1, TGFβ-activated kinase 1; TAB1/2, TAK1-binding protein 1/2; TIR; Toll/interleukin-1 receptor domain; TLR, Toll-like receptor; TRAF6, TNF receptor-associated factor 6.

The aberrant activation of innate immune signaling cascades represents one mechanism to drive constitutive activation of NF-κB signaling in lymphoid malignancies [10]. B cells express TLRs which recognize a wide variety of pathogen-associated molecular patterns (PAMPs) derived from bacteria, viruses, or fungi independently of the B-cell receptor (BCR) [26]. Structurally, TLRs are characterized by a conserved Toll/IL-1 receptor (TIR) domain which undergoes a conformational change after receptor ligation, providing a platform for the interaction with cytoplasmic TIR domain-containing proteins, such as the adapter protein myeloid differentiation primary response protein 88 (MyD88) [27]. MyD88 comprises an N-terminal death domain which is connected to a C-terminal TIR domain by a linker region [28]. Ligand binding results in dimerization of the TLRs and subsequent recruitment of MyD88 homodimers via TIR–TIR interactions [29,30]. MyD88 forms a high-molecular weight signaling complex, the so-called Myddosome, through a series of sequential interactions (Figure 1): First, MyD88 oligomerizes and recruits the IL-1R-associated kinases 1, 2, and 4 (IRAK1, 2, and 4) via a homotypic interaction involving their death domain. In the Myddosome, IRAK4 is activated by auto-phosphorylation and in turn phosphorylates IRAK1 [28,31,32,33,34]. IRAK2 can functionally substitute IRAK1, implicating that IRAK1 and IRAK2 are redundant for downstream signaling [35]. Once IRAK1 is fully phosphorylated, it dissociates from the receptor complex and activates the E3 ubiquitin ligase TNF receptor-associated factor 6 (TRAF6). TRAF6-dependent lysine 63 (K63)-linked polyubiquitination of itself and several other proteins facilitates the recruitment of the IKK complex and TGFβ-activated kinase 1 (TAK1) via the ubiquitin binding domains of the regulatory subunit IKKγ and the adapter proteins TAK1-binding protein (TAB), respectively [36,37,38]. TAK1-dependent phosphorylation activates IKKβ which in turn induces the proteasomal degradation of the inhibitory protein IκBα, thus triggering canonical NF-κB activation [3,39].

Recurrent oncogenic mutations of the adapter protein MyD88 have been identified in a variety of B-cell malignancies. As approximately 40% of ABC DLCBL biopsies harbor MyD88 mutations, MyD88 represents the most frequently mutated oncogene in this tumor entity [40]. Whereas different somatic mutations of MyD88 have been reported, the most prevalent missense mutation encodes the amino acid substitution L265P within the TIR domain [40]. The L265P mutation of MyD88 occurs at a high frequency in ABC DLBCL (30% of cases) and in Waldenström’s macroglobulinemia (WM; 90%) as well as less commonly in marginal-zone lymphoma (13%), gastric MALT lymphoma (9%), and CLL (3%) [40,41,42,43,44]. In contrast, gain-of-function mutations of MyD88 are rare or absent in other DLBCL subtypes, i.e., germinal center B cell-like (GCB) DLBCL and PMBL [40,45]. Ectopic expression of MyD88^L265P^, but not of wild-type MyD88 in GCB DLBCL cell lines, which exhibit *per se* little to no NF-κB activity, potently induces NF-κB activation, demonstrating the oncogenic capacity of this MyD88 variant. This gain-of-function has been attributed to the ability of MyD88^L265P^ to spontaneously oligomerize and thus activate IRAK1 and IRAK4 independently of a TLR ligand (Figure 1) [40,46]. In mice, expression of MyD88^L252P^ (the orthologous position of the human L265) is sufficient to trigger the formation of lymphoma morphologically resembling the ABC DLBCL phenotype [47]. Interestingly, the L265P mutant TIR domain is able to recruit endogenous wild-type MyD88 to trigger downstream signaling in vitro [46,48,49]. Whereas the kinase activity of IRAK1 is dispensable for the capacity of mutant MyD88 to promote the survival of ABC DLBCL, NF-κB activation driven by oncogenic MyD88 mutations critically relies on the kinase activity of IRAK4, implicating this kinase as an interesting therapeutic target in lymphoid malignancies [40,50,51]. Indeed, the highly selective IRAK4 inhibitors ND-2158 and ND-2110 abrogate aberrant NF-κB activation induced by oncogenic MyD88^L265P^ and thus efficiently suppress the growth of ABC DLBCL cells in vitro and in vivo [50].

Similar to the requirement of chronically active BCR signaling in B-cell malignancies (discussed in Section 3), the importance of TLR-derived signals in lymphomagenesis is under debate. On the one hand, expression of a non-functional variant of Unc93b1, which is required for the endolysosomal localization of TLR3, 7, and 9, as well as TLR9 deficiency block the proliferation of primary B cells induced by the expression of ectopic MyD88^L265P^ in vitro, implicating a continued dependence on upstream TLR9 activation [52]. On the other hand, in vivo depletion of TLR9 in mice rather suggests an inhibitory role of TLRs in MyD88^L265P^-transduced B cells [53]. The exact molecular and functional consequences of TLRs in MyD88^L265P^-mutated tumor cells need to be addressed in future studies, especially since this could have implications for the use of TLR agonists/antagonists in lymphoma therapy.

## 3. Chronic B-Cell Receptor Signaling

**Figure 2:**
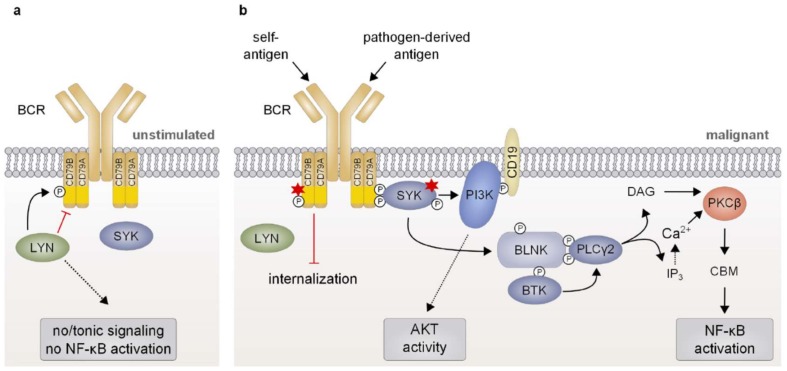
Mechanisms of chronic active BCR signaling in lymphoid malignancies. (**a**) In the absence of its cognate antigen, proximal BCR signaling remains inactive, and thus the protein tyrosine kinase SYK is not recruited to the co-receptors CD79A and CD79B. The Src kinase LYN prevents hyperactivation of BCR signaling by initiation of a negative feedback loop involving CD22 phosphorylation and subsequent activation of the phosphatase SHP-1. Tonic BCR signaling promotes B-cell survival via the PI3K/AKT pathway in an antigen-independent manner; (**b**) Engagement of the BCR, for instance by self-antigens or pathogen-derived antigens, results in receptor clustering and the induction of an intracellular signaling cascade. The subsequent phosphorylation of tyrosine residues in the ITAM regions of CD79A/B by Src kinases (e.g., LYN) allows the recruitment of SYK which in turn phosphorylates the adapter protein BLNK and thus promotes the formation of a proximal signaling complex involving BTK and PLCγ2. While activation of the AKT signaling axis is achieved by the SYK-mediated phosphorylation of CD19 and subsequent recruitment of PI3K, PLCγ2 activity generates the second messengers DAG and IP_3_ the latter triggering the influx of Ca^2+^ into the cell. DAG and elevated Ca^2+^ levels activate PKCβ which induces activation of canonical NF-κB through the CBM signalosome. Whereas overexpression of SYK augments NF-κB activation in some lymphomas, mutations in the co-receptors CD79A/B prevent the internalization of activated BCRs and thus promote chronic BCR signaling. Proteins that are affected by recurrent genetic lesions in lymphoid malignancies are denoted with a red asterisk. BCR, B-cell receptor; BLNK, B-cell linker protein; BTK, Bruton’s tyrosine kinase; CBM, CARMA1/BCL10/MALT1; DAG, diacylglycerol; IP_3_, inositol 1,4,5-trisphosphate; ITAM, immunoreceptor tyrosine-based activation motif; PI3K, phosphoinositide 3-kinase; PKCβ, protein kinase Cβ; PLCγ2, phospholipase Cγ2; SHP-1, Src homology 2 domain phosphatase 1; SYK, spleen tyrosine kinase.

The B-cell receptor complex comprises an immunoglobulin molecule (IgA, IgD, IgE, IgG, or IgM), which is anchored in the plasma membrane via a transmembrane domain, and a disulfide-linked CD79A/CD79B heterodimer essential for signal transmission. While recognition of the cognate antigen is achieved by the variable regions of the immunoglobulin chains (V_H_ and V_L_, respectively), CD79A/B contain immunoreceptor tyrosine-based activation motifs (ITAMs) within their cytoplasmic domains which are essential for the initiation of an intracellular signaling cascade in response to receptor engagement (Figure 2a) [54,55]. Ligand binding induces BCR clustering and phosphorylation of two invariant tyrosine residues within the ITAMs of CD79A/B by the Src family tyrosine kinase LYN (Figure 2b) [56,57]. Subsequently, spleen tyrosine kinase (SYK) is recruited to the phosphorylated ITAMs via its SH2 domain, resulting in SYK auto-phosphorylation as well as phosphorylation of several downstream mediators, such as CD19, Bruton’s tyrosine kinase (BTK) and B-cell linker protein (BLNK) [58]. Whereas CD19 phosphorylation leads to the recruitment of phosphoinositide 3-kinase (PI3K) culminating in activation of the AKT signaling axis, BLNK serves as a scaffold that binds both phospholipase Cγ2 (PLCγ2) and BTK, resulting in the BTK-dependent activation of PLCγ2 [59,60]. In turn, PLCγ2 converts phosphatidylinositol 4,5-bisphosphate (PIP_2_) to generate the second messengers inositol 1,4,5-trisphosphate (IP_3_) and diacylglycerol (DAG). Together, DAG and an increase in the intracellular Ca^2+^ levels induced by the action of IP_3_ activate the protein kinase Cβ (PKCβ), which subsequently phosphorylates the scaffold protein caspase recruitment domain (CARD) membrane-associated guanylate kinase (MAGUK) protein 1 (CARMA1; discussed in Section 4), thus triggering downstream NF-κB activation [61].

Due to its capacity to induce NF-κB activation, BCR signaling plays an important role in the survival and proliferation of a subset of B-cell malignancies [21,62]. Accordingly, it has been reported that chronic infections with viral and bacterial pathogens are often associated with lymphoma development due to persistent antigen-driven activation and proliferation of the B cells (Figure 2b) [63]. Several foreign antigens, for instance derived from hepatitis C virus, have been reported to be associated with certain types of lymphoma and most likely govern lymphoma proliferation and survival in a BCR-dependent manner [63,64]. In contrast, the ABC subtype of DLBCL seems to rely on chronic BCR signaling driven by self-antigens, since the survival of ABC DLBCL cell lines is impaired upon substitution of the IgH variable region of their BCRs [65]. Interestingly, the BCRs of some ABC DLBCL cell lines are reactive towards self-antigens present in apoptotic debris or towards an invariant part of its own V region [65]. The toxicity caused by knockdown of the essential BCR subunits CD79A/B or of downstream signaling effectors, such as BTK, SYK, and PLCγ2, observed in most ABC DLBCL cells further corroborates the notion that these lymphoid tumors critically rely on chronic active BCR signaling [66,67]. Conversely, the GCB subtype of DLBCL is independent of chronic BCR activation but instead requires “tonic”, antigen-independent BCR signals which promote survival by activating the PI3K/AKT pathway [66,68,69,70]. In line with chronic BCR triggering, cell lines as well as biopsies of ABC DLBCL typically exhibit BCR clustering on the cell surface, which correlates with increased levels of tyrosine phosphorylation and indicates sustained BCR signaling [67]. Chronic BCR signaling also plays a crucial role in CLL, since 30% of this cancer entity express a similarly rearranged BCR using a distinct subset of V_H_, D_H_, and J_H_ gene segments, which can also be found in ABC DLBCL [65,71]. These so-called “stereotyped” BCRs are thought to respond to similar antigens, most likely presented by the tumor itself, such as proteins of apoptotic cells or an epitope of the BCR [72,73,74]. Interestingly, expression of a CLL-derived IgH V region has been shown to sustain the survival of an ABC DLBCL cell line, suggesting that a similar (self-) antigen is driving chronic BCR signaling in a subset of CLL and ABC DLBCL [65].

To maintain chronic BCR activation, approximately 20% of ABC DLBCL tumors exhibit somatic mutations in the co-receptors CD79B and less commonly CD79A (Figure 2b) [67]. A frequently occurring missense mutation present in 18% of ABC DLBCL biopsies involves the substitution of the membrane-proximal ITAM tyrosine (Y196) of CD79B. These CD79B mutations are associated with increased surface expression of the BCR in the context of chronic active BCR signaling and with a reduced activation of the tyrosine kinase LYN, which plays a dual role in BCR signaling [67,75]. Besides its positive regulatory role in the initial tyrosine phosphorylation cascade, LYN also exerts inhibitory effects on BCR-induced signaling. On the one hand, by phosphorylation of inhibitory motifs in CD22, LYN mediates the recruitment and activation of SHP-1, a phosphatase that quenches BCR signaling by the removal of ITAM phosphorylation [76,77,78,79,80]. On the other hand, LYN activity has been shown to promote BCR internalization, suggesting that reduced LYN activation in CD79-mutated ABC DLBCLs results in an increased surface expression of chronically activated BCRs [67]. A recent study has further highlighted the importance of *CD79B* mutations for surface expression of the BCR in ABC DLBCL cells and provided a rationale for the frequently observed co-occurrence of *CD79B* and *MYD88* mutations in B-cell malignancies [40]: While *CD79B* mutations alone are not sufficient to enhance NF-κB-mediated B-cell proliferation and *MYD88* mutations on their own decrease surface IgM/BCR expression reminiscent of anergic B cells, the combination of *CD79B* and *MYD88* mutations cooperates in plasmablastic differentiation [81]. Collectively, CD79 mutations sustain high BCR levels at the cell surface despite chronic BCR activation and thus prolong BCR-dependent signaling. It is tempting to speculate that the gene loss of LYN occurring in 60% of patients suffering from WM might, similar to the CD79A/B mutations, sustain surface BCR expression and thus chronic BCR signaling [82]. Additionally, overexpression of SYK as observed in MCL and peripheral T-cell lymphomas most likely also contributes to NF-κB activation, even though the molecular details have not been investigated so far [83,84].

From a clinical perspective, a multitude of new inhibitors targeting chronic BCR signaling are in the pipeline or already approved for the treatment of particular lymphoid malignancies [85]. Several of these inhibitors target BTK, such as ibrutinib or acalabrutinib, which are currently used or have been proposed for the treatment of CLL, MCL, WM, and DLBCL [86,87,88,89,90]. Other inhibitors targeting kinases involved in the proximal tyrosine phosphorylation cascade, such as SYK and LYN, may be able to potently reduce NF-κB activation caused by chronic BCR signaling [67]. While SYK can be targeted with fostamatinib [85,91,92], the small-molecule inhibitor dasatinib, which was initially utilized as an inhibitor of the oncogenic BCR-ABL fusion protein in chronic myelogenous leukemia, was shown to inhibit also LYN and BTK in CLL [93,94,95].

## 4. Genetic Alterations Driving Constitutive NF-κB Activation via the CBM Signalosome

**Figure 3:**
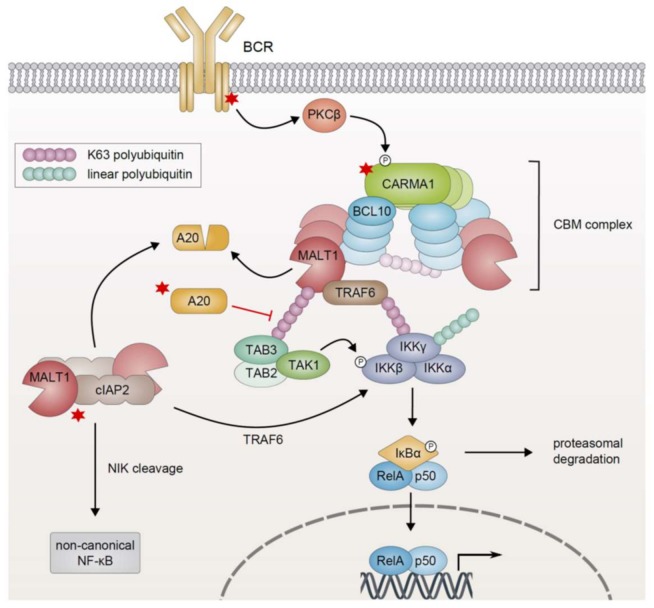
Increased activity of the CARMA1/BCL10/MALT1 signalosome drives constitutive NF-κB activation. Antigen-dependent triggering of the BCR results in the activation of PKCβ, which phosphorylates the scaffold protein CARMA1 at several residues within its inhibitory linker region. Activated CARMA1 in turn nucleates the formation of a fibrillary high-molecular weight signaling complex (CBM complex) comprising long BCL10 filaments and MALT1. Subsequently, the E3 ubiquitin ligase TRAF6 is recruited to the CBM complex via an interaction with MALT1 and catalyzes the K63-linked polyubiquitination of MALT1, BCL10, and itself. These K63-linked polyubiquitin chains as well as the linear polyubiquitin chains generated by the action of the LUBAC allow the recruitment and activation of the IKK complex. TAK1 which is recruited to the signaling complex via the ubiquitin binding domains of its accessory proteins TAB2 and TAB3 promotes IKK activation by phosphorylating IKKβ. IKK activity in turn results in the proteasomal degradation of IκBα and the subsequent nuclear translocation of transcriptionally active NF-κB heterodimers. In lymphoid malignancies, chronically active BCR signaling as well as gain-of-function mutations of the scaffold protein CARMA1 can lead to hyperactivation of the CBM signalosome and thus induce constitutive NF-κB activity. Genetic inactivation of A20, which negatively regulates IKK activity by deubiquitination of activated signaling effectors, augments NF-κB activation in several lymphoid cancers. Additionally, A20 can be proteolytically inactivated by the MALT1 protease, which is activated in the framework of the CBM complex. Expression of the oncogenic cIAP2-MALT1 fusion protein induces activation of canonical NF-κB in a TRAF6-dependent manner. The constitutive protease activity of cIAP2-MALT1 further promotes NF-κB activation by proteolytic inactivation of A20. Additionally, NIK cleavage by cIAP2-MALT1 also promotes non-canonical NF-κB activity. Proteins that are affected by recurrent genetic lesions in lymphoid malignancies are denoted with a red asterisk. BCL10, B-cell lymphoma 10; BCR, B-cell receptor; CARMA1, caspase recruitment domain membrane-associated guanylate kinase protein 1; cIAP2, cellular inhibitor of apoptosis protein 2; IκB, inhibitor of κB; IKK, IκB kinase; LUBAC, linear ubiquitin chain assembly complex; MALT1, mucosa-associated tissue lymphoma translocation protein 1; NIK, NF-κB inducing kinase; PKCβ, protein kinase Cβ; TAK1, TGFβ-activated kinase 1; TAB2/3, TAK1-binding protein 2/3; TRAF6, TNF receptor-associated factor 6.

In lymphocytes, the activation of canonical NF-κB signaling in response to antigen receptor ligation requires the formation of a multimeric signaling module, termed the CARMA1/BCL10/MALT1 (CBM) complex, which functionally links antigen receptor proximal signaling events with the activation of the IKK complex [96,97,98]. Antigen binding to the BCR triggers a signaling cascade involving the activation of Src kinases and ultimately leads to the activation of PKCβ (discussed in Section 3), which in turn phosphorylates the scaffold protein CARMA1 within the flexible linker region situated between its coiled-coil and C-terminal MAGUK domain (Figure 3) [99,100]. In quiescent lymphocytes, CARMA1 adopts an auto-inhibited, inactive conformation, which is stabilized by an intramolecular interaction between its inhibitory linker and the region spanning the CARD and coiled-coil domain. PKC-mediated phosphorylation activates CARMA1 by triggering a conformational change that facilitates the oligomerization of CARMA1 via the coiled-coil domain [99,100,101,102]. Subsequently, active CARMA1 nucleates the formation of long B-cell lymphoma 10 (BCL10) filaments through CARD–CARD-mediated homotypic interactions [103,104,105,106]. The mucosa-associated lymphoid tissue lymphoma translocation protein 1 (MALT1) is recruited to the fibrillary signaling complex due to its constitutive association with BCL10 [107,108,109]. The assembly of a high-molecular weight complex comprising CARMA1, oligomerized BCL10, and MALT1 is indispensable for NF-κB activation in response to antigen receptor ligation and represents a hallmark of lymphocyte activation [61,102,110]. Within the CBM complex, MALT1 recruits the ubiquitin ligase TRAF6, which in turn mediates polyubiquitination of MALT1, BCL10, and itself (Figure 3). These polyubiquitin chains serve as docking sites for the physical recruitment of the IKK complex via the ubiquitin binding motif of the regulatory subunit IKKγ [111,112,113,114]. Additionally, the linear ubiquitin chain assembly complex (LUBAC) is recruited to the CBM signalosome and in turn promotes activation of the IKK complex by mediating the linear ubiquitination of IKKγ [115,116,117]. Polyubiquitination also results in the recruitment of TAK1 via the ubiquitin binding domain of the adapter proteins TAK-binding protein 2/3 (TAB2/3). Collectively, the CBM complex serves as a signaling platform which facilitates the activation of the IKK complex through a series of ubiquitination-dependent interactions, culminating in TAK1-induced phosphorylation of the catalytic subunit IKKβ [39,112,113,118].

In activated lymphocytes, the paracaspase MALT1 plays a dual role in promoting antigen-induced NF-κB activation: As a scaffold protein in the framework of the CBM signalosome, MALT1 on the one hand facilitates the physical recruitment and activation of the IKK complex by providing binding sites for the ubiquitin ligase TRAF6. On the other hand, the protease activity of MALT1 further potentiates pro-inflammatory signaling in response to antigen receptor stimulation [114]. The central protease domain of MALT1 shares homology with proteases of the caspase and metacaspase family and contains conserved cysteine and histidine residues essential for its catalytic activity [107,119]. In contrast to caspases, which catalyze substrate cleavage after the negatively charged amino acid aspartate, MALT1 cleaves its target proteins after positively charged arginine residues [61,120,121]. MALT1 dimerization via its protease domain is essential for the acquisition of a catalytically active conformation, occurs in the context of CBM complex assembly and is promoted by mono-ubiquitination of MALT1 [103,104,122,123,124]. Intriguingly, MALT1 protease activity potentiates and sustains NF-κB activation in an IKK-independent manner, presumably by the proteolytic inactivation of A20 and RelB [121,125]. MALT1-dependent cleavage and subsequent proteasomal degradation of RelB, which impedes classical NF-κB1 activation by the formation of transcriptionally inactive RelA/RelB heterodimers and/or by competing for DNA binding sites, results in enhanced DNA binding of RelA and c-Rel [125,126]. Additionally, proteolytic inactivation of the deubiquitinating enzyme A20, which removes K63-linked polyubiquitin chains from key signaling mediators, such as TRAF6, IKKγ, and MALT1, and thus negatively regulates NF-κB activation, sustains maximum NF-κB induction (Figure 3) [121,127]. Auto-processing of MALT1 is assumed to also be essential for NF-κB activation in lymphocytes, although the molecular mechanism of this contribution remains unclear thus far [128]. In contrast to the role of MALT1 catalytic activity in promoting NF-κB activation, MALT1-dependent cleavage of heme-oxidized iron-responsive element-binding 2 ubiquitin ligase-1 (HOIL-1), a component of LUBAC that promotes IKKγ ubiquitination and thus activation of the IKK complex, instead dampens NF-κB signaling and might be involved in the termination of CBM/IKK-mediated NF-κB activity [129,130,131].

While CBM-mediated NF-κB activation plays a critical role in lymphocyte proliferation and loss-of-function mutations result in immunodeficiency, aberrant constitutive NF-κB activation is not only associated with autoimmune diseases but also with the development of lymphoid malignancies [98,132,133]. Recurrent gain-of-function mutations in the genes encoding CBM proteins or their upstream regulators result in constitutive CBM-dependent NF-κB activation and have been detected in a wide range of lymphoid malignancies including ABC DLBCL, MCL, MALT lymphoma, acute T-cell leukemia/lymphoma (ATLL), and Sézary syndrome [16,23,134,135]. The toxicity of RNAi-mediated silencing of either CARMA1, BCL10 or MALT1 expression observed in ABC DLBCL cell lines further demonstrates the importance of CBM-mediated signaling in this tumor entity [66]. Hyperactivity of the CBM signalosome associated with gain-of-function mutations of the central scaffold protein CARMA1 or its upstream regulators (e.g., CD79A/B, discussed in Section 3) as well as with constitutive BCR signaling driven by self-antigens has emerged as a hallmark of lymphomagenesis [65,67,98,136,137].

### 4.1. Oncogenic CARMA1 Mutations

Oncogenic CARMA1 mutations driving constitutive signaling activity of the CBM complex have initially been discovered in approximately 10% of patients suffering from the aggressive ABC subtype of DLBCL which relies on constitutive NF-κB signaling for survival and proliferation [137]. At present, several tumor entities including ABC DLBCL, ATLL, and Sézary syndrome as well as a congenital B-cell lymphocytosis, a B cell proliferative syndrome associated with an increased risk of lymphoma development, have been found to harbor activating missense mutations of CARMA1 [135,138,139,140]. The majority of the identified somatic gain-of-function mutations of CARMA1 are located in the proximity of or within the region spanning the CARD and the coiled-coil domain. Mechanistically, these mutations are thought to disrupt auto-inhibition of CARMA1, thus favoring oligomerization and activation of downstream signaling (Figure 3) [137]. Indeed, ectopic expression of these CARMA1 mutants has been shown, one the one hand, to drive constitutive activation of CBM-mediated NF-κB signaling independently of upstream signals and, on the other hand, to potentiate NF-κB activation in response to antigen receptor stimulation [137].

### 4.2. Overexpression of BCL10/MALT1 and cIAP2-MALT1 Fusion Protein

Another tumor entity critically relying on constitutive CBM signaling is represented by lymphomas of the mucosa-associated lymphoid tissue, which occur most commonly in the stomach typically due to chronic infection, e.g., with *Helicobacter pylori*, and can be successfully treated at early stages by eradication of the source of inflammation [134,141,142]. At advanced stages, however, these MALT lymphomas are associated with distinctive chromosomal translocations, which either lead to the overexpression of BCL10 and MALT1 [143,144,145] or result in the generation of a constitutively active fusion protein comprising the N-terminal part of cellular inhibitor of apoptosis protein 2 (cIAP2, also known as API2) and the C-terminus of MALT1 [24,146,147]. The cIAP2-MALT1 fusion protein is able to auto-oligomerize independently of BCL10 and upstream signals via the baculovirus inhibitor of apoptosis repeat (BIR) domain of the cIAP2 moiety which binds heterotypically to the C-terminal region of MALT1 (Figure 3) [134,148,149]. Constitutive MALT1 protease activity and the capacity of cIAP2-MALT1 to potently activate both the canonical NF-κB1 (via the MALT1-dependent recruitment of TRAF6 and proteolytic inactivation of A20) and non-canonical NF-κB2 pathways (discussed in Section 5) drive the growth of these MALT lymphomas [24,107,121,148,149].

Aberrant induction of MALT1 protease activity is a major consequence of constitutive CBM signaling and has been reported to be essential for the survival of several lymphoid malignancies, such as ABC DLBCL, MCL, and CLL, making MALT1 an attractive therapeutic target in lymphoma treatment [150,151,152,153]. Even though at present no MALT1 inhibitors have entered the clinic, high-throughput screening revealed several small-molecule inhibitors targeting MALT1 protease activity [154,155,156]. Indeed, pharmacological inhibition of the MALT1 protease function has been reported to exert selective toxicity towards MALT1-dependent lymphomas both in vitro and in vivo using xenograft mouse models [154,155].

### 4.3. Inactivation of TNFAIP3/A20

In addition to constitutive signaling via the CBM complex, aberrant NF-κB activity in lymphoid malignancies can also be promoted by the genetic inactivation of A20, which negatively regulates IKK activation most likely by removing the K63-linked polyubiquitin chains from the activated CBM signalosome (Figure 3) [127,157,158,159]. Indeed, at least one allele of *TNFAIP3* encoding A20 is frequently targeted by mutations, deletions, or epigenetic silencing which result in a partial or complete loss of its negative regulatory function in several lymphoid malignancies, such as HL (approximately 45% of cases), PMBL (30%), ABC DLBCL (25%), and MALT lymphoma (20%) [160,161,162,163]. Recent reports suggest that loss of A20 function on its own might not result in sufficient NF-κB activation to support lymphomagenesis [164]. Instead, inactivation of A20 is often found to be associated with additional genetic aberrations, such as *MYD88* or *CD79A/B* mutations, which drive constitutive NF-κB activation [40,62]. The role of A20 as a tumor suppressor in B-cell lymphoma is further supported by the toxicity of ectopic expression of A20 in A20-deficient ABC DLBCL cell lines [160,161].

### 4.4. LUBAC Polymorphism

Genetic analyses of lymphomas have recently identified rare germ line polymorphisms which are enriched in ABC DLBCL patients and promote polyubiquitin-dependent NF-κB activation. These SNPs cause amino acid substitutions in HOIL-1 interacting protein (HOIP, encoded by *RNF31*), promote its interaction with HOIL-1 and result in an increased activity of the LUBAC [165]. Interestingly, RNAi-mediated silencing of LUBAC expression or inhibition of its activity have been reported to reduce constitutive NF-κB activity and to thus induce cell death in ABC DLBCL cells, suggesting an important role of linear ubiquitin in oncogenic NF-κB activation in these lymphomas [165,166].

## 5. Constitutive Activation of Non-Canonical NF-κB Signaling

**Figure 4:**
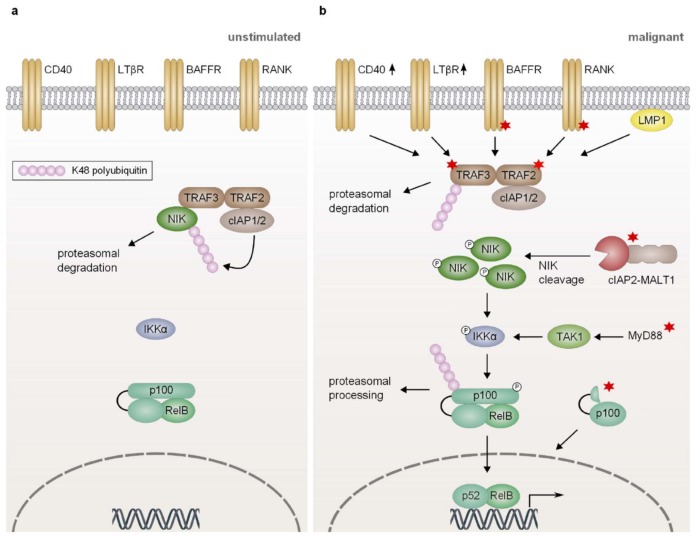
Genetic lesions driving non-canonical NF-κB activation in lymphoid malignancies. (**a**) In unstimulated cells, TRAF3 recruits NIK to an E3 ubiquitin ligase complex comprising TRAF2 and cIAP1/2 which constantly marks NIK for proteasomal degradation by K48-linked polyubiquitination. The heterodimer of RelB and the p52 precursor protein p100 is sequestered in the cytoplasm; (**b**) Activated or mutated members of the TNF receptor family, such as CD40, BAFF-R, LTβR, and RANK or the EBV-encoded CD40 mimic LMP1, recruit the complex consisting of TRAF3, TRAF2, and cIAP1/2 to the cell membrane and induce the proteasomal degradation of TRAF3. In the absence of TRAF3, NIK is released from the inhibitory E3 ubiquitin ligase complex and accumulates in the cytoplasm where it subsequently phosphorylates and activates IKKα. In turn, IKKα phosphorylates the precursor protein p100, thus targeting it for processing by the proteasome. Proteasomal processing of p100 generates p52 and results in the nuclear translocation of transcriptionally active p52/RelB heterodimers. Enhanced expression of NIK due to copy number gains and loss-of-function mutations of TRAF2, TRAF3, or cIAP2 result in increased NIK protein levels driving non-canonical activation of NF-κB in lymphoid malignancies. Similarly, proteolytic cleavage by the constitutively active cIAP2-MALT1 fusion protein stabilizes NIK by removal of the TRAF3-binding site. Additionally, oncogenic MyD88 mutations can promote the activation of IKKα in a TAK1-dependent manner. In certain lymphomas aberrant activation of non-canonical NF-κB is governed by the expression of a truncated, constitutively active version of the precursor p100 lacking the inhibitory ankyrin repeats. Proteins that are affected by recurrent genetic lesions in lymphoid malignancies are denoted with a red asterisk. BAFF-R, B cell-activating factor receptor; cIAP1/2, cellular inhibitor of apoptosis protein 1/2; EBV, Epstein-Barr virus; IKKα, IκB kinase α; LMP1, latent membrane protein 1; LTβR, lymphotoxin β receptor; MALT1, mucosa-associated tissue lymphoma translocation protein 1; MyD88, myeloid differentiation primary response protein 88; NIK, NF-κB inducing kinase; RANK, receptor activator of NF-κB; TAK1, TGFβ-activated kinase 1; TRAF2/3, TNF receptor-associated factor 2/3.

In resting lymphocytes, the non-canonical, also termed alternative, NF-κB pathway is inactive, as NF-κB inducing kinase, a central player in this pathway, is constitutively targeted for proteasomal degradation [4,167]. Degradation of NIK relies on K48-linked polyubiquitination catalyzed by the E3 ubiquitin ligases cIAP1 and cIAP2 which are recruited to NIK via an interaction with TRAF3 (Figure 4a) [4]. Upon activation of certain members of the TNF receptor superfamily, such as CD40 or the BAFF receptor (BAFF-R), the complex comprising TRAF3, TRAF2, and cIAP1/2 is recruited to the receptor, resulting in the cIAP1/2-dependent K48-linked polyubiquitination and subsequent proteasomal degradation of TRAF3 (Figure 4b) [4]. Depletion of TRAF3 abrogates the interaction between NIK and cIAP1/2, thus stabilizing NIK which in turn phosphorylates and activates IKKα [168]. Subsequently, IKKα phosphorylates the precursor protein p100 (NF-κB2) and thus marks it for processing by the proteasome to generate p52 which forms a transcriptionally active heterodimer with RelB [168].

Several lymphoid cancers, particularly multiple myeloma but also Hodgkin lymphoma and cIAP2-MALT1 expressing MALT lymphoma (discussed in Section 4), harbor various genetic alterations which affect different regulators of the non-canonical NF-κB pathway and rely on constitutive nuclear activity of p52/RelB heterodimers [19,20,160,169,170]. Since activation of non-canonical NF-κB signaling is primarily regulated through the tight control of NIK protein levels, most of the genetic aberrations observed in lymphoid malignancies result in the increased expression or stabilization of NIK (Figure 4b). Indeed, increased NIK protein levels caused by copy number gains in the *MAP3K14* gene which encodes NIK or chromosomal translocations relocating *MAP3K14* into the proximity of immunoglobulin enhancer elements can be frequently observed in multiple myeloma and HL [20,170]. Similarly, a NIK fusion protein lacking the TRAF3-binding domain exhibits increased stability due to loss of TRAF3-dependent proteasomal degradation [19,20,171]. As an alternative oncogenic mechanism to augment NIK activity and thus non-canonical NF-κB activation, negative regulators of NIK protein stability are frequently inactivated by deletions, loss-of-function mutations or transcriptional silencing [9]. Whereas loss-of-function mutations of TRAF2 or cIAP2 have been described in MCL and DLBCL, inactivating mutations or homozygous deletions of the gene encoding TRAF3 have been reported in HL (15% of cases), DLBCL (15%), and in multiple myeloma (50%) [9,19,20,23,45,160,170,172].

Stabilization of NIK and consequently increased processing of p100 can also be achieved by overexpression or mutation of the receptors which induce non-canonical NF-κB activity in a stimulus-dependent manner in normal lymphocytes. Mechanistically, recruitment of TRAF3, TRAF2, and cIAP1/2 to activated or mutated TNF superfamily receptors, such as CD40, BAFF-R, RANK, and the lymphotoxin β receptor (LTβR), induces the cIAP1/2-dependent degradation of TRAF3 and promotes stabilization of NIK [19,20,160]. For instance, a missense mutation (H159Y) targeting the cytoplasmic tail of the BAFF-R identified in follicular lymphoma, DLBCL, and less commonly in MALT lymphoma results in the increased recruitment of TRAF2, TRAF3, and TRAF6 to the receptor [173]. Additionally, overexpression of the receptor CD40 resulting in enhanced p100 processing has been reported in rare cases of multiple myeloma [19,20]. Similarly, genetic alterations affecting LTβR and RANK have been reported in multiple myeloma and DLBCL [19,160].

Recently, oncogenic MyD88 mutations, such as L265P (discussed in Section 2), have been shown to induce the activation of NIK and thus increase processing of p100 and p105 in DLBCL (Figure 4b) [174]. Interestingly, p100 processing is required to maintain the ABC phenotype, since knockdown of p100 reduced the expression of genes, such as *IRF4* and *BCL6*, typically associated with the ABC subtype of DLBCL. Conversely, ectopic expression of MyD88^L265P^ in GCB DLBCL cell lines has been shown to trigger p100 processing in a TAK1- and IKKα-dependent manner and to alter the B-cell differentiation status towards a phenotype resembling ABC DLBCL [174]. Besides genetic alterations targeting important regulators of alternative NF-κB activation, latent infection with the Epstein-Barr virus (EBV) induces non-canonical NF-κB signaling by introduction of the latent membrane protein 1 (LMP1) in approximately 40% of HL cases [175,176]. LMP1 is highly homologous to the cytoplasmic domain of the TNF receptor CD40 and induces IKKα-dependent p100 processing via the spontaneous formation of signaling aggregates [177,178,179,180]. Similarly, NF-κB2 activation mediated by the protein Tax of the human T-cell leukemia virus type 1 (HTLV-1) is often associated with ATLL [181].

Furthermore, rearrangement or partial deletions within the *NFKB2* gene locus which disrupt the inhibitory ankyrin repeats at the C-terminus of the precursor p100 have been found to result in the generation of a truncated, constitutively active p100 protein (Figure 4b) [20,182,183]. Additionally, the deubiquitinase CYLD, which negatively regulates NF-κB activation by removing K63-linked polyubiquitin chains from IKKγ, TRAF2, and TRAF6, is frequently inactivated by deletion, mutation, or transcriptional silencing in multiple myeloma [19,20,184,185,186].

Approximately 25% of gastric MALT lymphomas harbor the chromosomal translocation t(11;18) which results in the expression of a cIAP2-MALT1 fusion protein retaining the proteolytic activity of MALT1 (discussed in Section 4.2) [146,147]. In addition to promoting canonical NF-κB signaling, the cIAP2-MALT1 fusion protein has also been found to potently induce activation of the non-canonical NF-κB pathway (Figure 3 and Figure 4b) [24,107]. Oligomerization of the fusion protein via the cIAP2 moiety is assumed to stimulate constitutive protease activity of MALT1 [24,148]. Additionally, the cIAP2 portion mediates the recruitment of NIK which is subsequently cleaved by the MALT1 protease [24]. Proteolytic cleavage removes the TRAF3-binding domain while leaving the kinase domain of NIK intact and thus generates a truncated, constitutively active NIK which is resistant to negative regulation by proteasomal degradation and promotes constitutive p100 processing [24].

Collectively, loss of TRAF3 function, enhanced degradation of TRAF3 or increased expression of NIK augments processing of p100 and thus the nuclear accumulation of transcriptionally active p52/RelB heterodimers. Increased NIK activity can also promote canonical NF-κB activation, since NIK is also able to activate IKKβ [19,20,187]. As aberrant NIK activity driving constitutive activation of both canonical and non-canonical NF-κB signaling constitutes a common consequence of most of the genetic aberrations found in a large subset of multiple myeloma and Hodgkin lymphoma patients, pharmacological NIK inhibition represents an attractive therapeutic strategy for the treatment of these tumor entities. However, even though some NIK inhibitors have been developed, so far none has entered the clinics [188,189].

## 6. Aberrant Expression of IκB Proteins

### 6.1. Classical IκB Proteins

**Figure 5:**
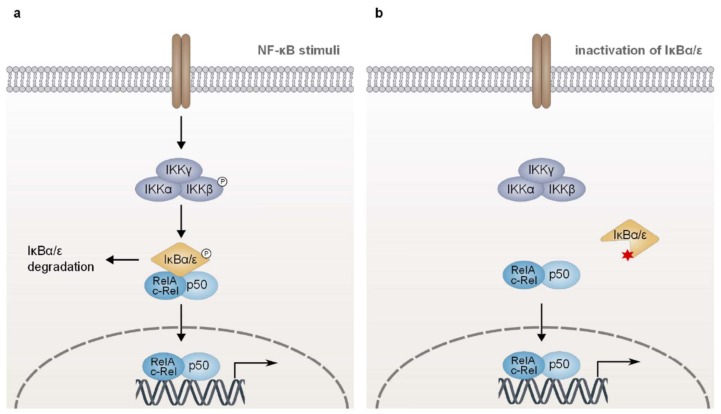
Inactivating mutations in classical IκB proteins promote canonical NF-κB activation. (**a**) The canonical NF-κB pathway is induced by the ligand-dependent activation of a variety of receptors, such as antigen receptors, the IL-1 receptor, Toll-like receptors and members of the TNF receptor family. Stimulus-dependent activation of the IKK complex results in the phosphorylation and subsequent proteasomal degradation of IκBα and IκBε. This allows the nuclear translocation of transcriptionally active RelA/p50 or c-Rel/p50 heterodimers; (**b**) Genetic alterations resulting in the expression of non-functional truncated versions of IκBα and IκBε, which lack part of the ankyrin repeat domain and are thus unable to bind to the NF-κB transcription factors, promote constitutive NF-κB activation in a subset of lymphoid malignancies. Proteins that are affected by recurrent genetic lesions in lymphoid malignancies are denoted with a red asterisk. IκB, inhibitor of κB; IKK, IκB kinase.

In non-activated cells, NF-κB dimers are sequestered in the cytoplasm by an interaction with classical IκB proteins (IκBα, IκBβ, and IκBε), which mask the nuclear localization signal (NLS) of the NF-κB subunits and thus prevent their nuclear translocation (Figure 5a). Stimulation-dependent proteasomal degradation of the IκB proteins allows the translocation of the NF-κB dimers to the nucleus, where they modulate the expression of a variety of genes [2,3]. The best characterized classical IκB protein, IκBα, is composed of a signal response domain, ankyrin repeats, a PEST domain as well as a nuclear export signal (NES) and binds preferentially RelA/p50 heterodimers. The presence of an NES suggests that besides its function as a cytoplasmic inhibitor, IκBα is also involved in the termination of NF-κB transcriptional activity by promoting both the dissociation of RelA/p50 complexes from the DNA and their subsequent nuclear export [3,190,191,192].

In lymphoid malignancies, such as HL or DLBCL, characteristic genetic aberrations targeting the classical IκB proteins can trigger NF-κB activation downstream of the IKK complex [193,194]. The malignant cellular entity in HL, the Hodgkin-Reed-Sternberg (HRS) cell, is present at a low frequency (<1% of the tumor), while the bulk of the tumor is formed by activated inflammatory cells [195]. Aberrant NF-κB activation in these malignant cells is not only driven by the inflammatory tumor microenvironment or by latent infection with EBV, but also by somatic mutations of key NF-κB regulators, such as classical IκB proteins [10,196,197,198]. In HL, various genetic lesions have been described that result in the generation of truncated IκBα isoforms which lack part of the ankyrin repeats and are thus unable to sequester the NF-κB dimers in the cytoplasm (Figure 5b) [193,194,199,200]. Interestingly, inactivating mutations of IκBα have been detected preferentially in EBV-negative cases of HL (approximately 20% of cases; discussed in Section 5), suggesting that inactivation of IκBα is selected for as an alternative strategy to sustain NF-κB activation [194,197,201]. Besides HL, mutations negatively affecting the function of IκBα have been reported, albeit at lower frequency, in MALT lymphoma and in DLBCL, similarly providing an alternative mechanism for NF-κB activation in these tumor entities [202,203,204].

Analogous to inactivation of IκBα, loss-of-function mutations of IκBε, which result for instance in the expression of truncated versions lacking the ankyrin repeats essential for the interaction with NF-κB dimers, have been reported in HL, CLL, and PMBL as well as at a lower frequency in DLBCL and MCL (Figure 5b) [205,206,207,208]. Mechanistically, IκBε is assumed to limit the nuclear localization of Rel-containing dimers in a manner equivalent to IκBα [206,209,210]. While IκBα, however, predominantly regulates the cytoplasmic sequestration of RelA/p50 heterodimers, IκBε preferentially binds to c-Rel homodimers and c-Rel/p50 complexes [206,209]. Physiologically, stimulated B cells of IκBε-deficient mice exhibit increased proliferation and survival due to enhanced NF-κB activity [206,211]. Collectively, loss-of-function mutations targeting the classical IκB proteins IκBα and IκBε contribute to sustained NF-κB activation in lymphoid malignancies, indicating an important role of these negative regulators as tumor suppressors.

In lymphoid malignancies that rely on constitutive NF-κB activation but express functionally intact IκB proteins, preventing the proteasomal degradation of the IκB proteins constitutes an attractive therapeutic strategy. The proteasome inhibitor bortezomib is able to block the degradation of the classical IκB proteins and has been approved for multiple myeloma therapy, although it remains unclear if the beneficial effect of bortezomib can be attributed solely to NF-κB inhibition [212,213,214]. In clinical trials, bortezomib has also been shown to improve the efficacy of chemotherapy in ABC DLBCL [215].

### 6.2. Atypical IκB Proteins

**Figure 6:**
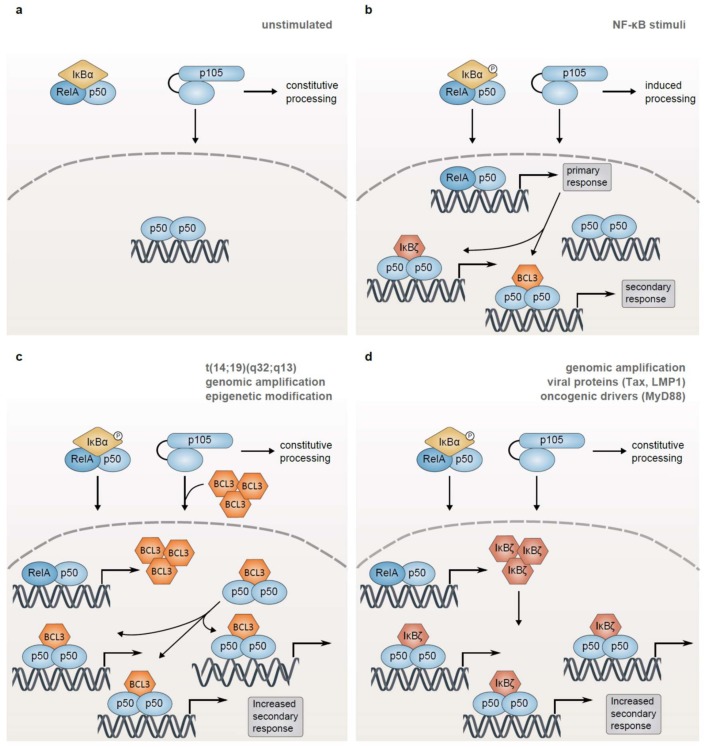
Abnormal expression of the atypical IκB proteins BCL3 and IκBζ promotes NF-κB activation in lymphoid malignancies. (**a**) In resting cells, DNA-bound homodimers of the NF-κB subunit p50 can be found in the nucleus due to constitutive processing of the p50 precursor p105; (**b**) Upon B-cell stimulation, the activated IKK complex induces both the generation of p50 by proteasomal processing of its precursor p105 and the proteasomal degradation of the inhibitor IκBα. This leads to the nuclear translocation of RelA/p50 heterodimers which drive the transcription of primary response genes, such as *BCL3* and *NFKBIZ*. In turn, BCL3 and IκBζ modulate the secondary NF-κB response by binding to DNA-bound p50 homodimers in the nucleus; (**c**) The chromosomal translocation t(14;18)(q32;q13) juxtaposing *BCL3* with the *IGH* locus, genomic amplifications, or epigenetic modifications result in enhanced expression of BCL3, which in turn increases the nuclear translocation of p50 and stabilizes DNA-bound p50 homodimers, thus augmenting the secondary NF-κB response; (**d**) Genomic amplification of the *NFKBIZ* locus as well as the expression of oncogenic MyD88^L265P^ or the viral proteins Tax and LMP1 leads to increased expression of IκBζ in a subset of lymphoid tumors. IκBζ promotes the secondary NF-κB response through binding to p50 homodimers in the nucleus. BCL3, B-cell lymphoma 3; *IGH*, immunoglobulin heavy chain (locus); IκB, inhibitor of κB; IKK, IκB kinase; LMP1, latent membrane protein 1; MyD88, myeloid differentiation primary response protein 88.

Not only classical IκB proteins are targeted by genetic alterations in lymphoid malignancies, but also the expression of the so-called atypical IκBs including BCL3 and IκBζ can be affected in these cancers. Unlike the classical IκB proteins, atypical IκBs are not regulated by IKK phosphorylation and proteasomal degradation, but rather by their inducible expression. While atypical IκB proteins are generally not expressed in quiescent cells, they are strongly induced in the primary response upon NF-κB activation (Figure 6a,b) [5,7]. In contrast to their classical relatives, atypical IκBs interact with NF-κB proteins predominantly in the nucleus. Although the atypical IκB proteins were initially defined as NF-κB inhibitors, it is by now well established that they can act also as co-activators for a particular set of target genes [5,7]. Several studies have reported the importance of atypical IκB proteins in immune homeostasis and there is growing evidence for an involvement of these transcriptional regulators in the pathogenesis of lymphoid malignancies.

B-cell lymphoma 3 (BCL3) was first identified as a proto-oncogene in patients suffering from B-cell chronic lymphocytic leukemia [216,217,218]. Structurally, BCL3 is characterized by a conserved NLS and two transactivation domains (TAD) encompassing seven ankyrin repeats that mediate binding to NF-κB proteins [7,219,220,221]. Through an interaction with p50 or p52 homodimers, BCL3 can act both as an activator and as a repressor of NF-κB target gene transcription. How this dual function of BCL3 is realized on a molecular level remains unclear. It has been reported, on the one hand, that BCL3 is able to stabilize transcriptionally repressive p50 or p52 homodimers, and, on the other hand, that it binds to p50 or p52 homodimers and induces the transcription of target genes via its transactivation domains [221,222]. One explanation for the opposing BCL3 effects could lie in its capability to recruit both co-activator and co-repressor complexes comprising chromatin modifiers, such as p300 or HDAC1, respectively, to DNA-bound p50 and p52 homodimers in a context-dependent manner [223,224,225].

Different genetic aberrations affecting the NF-κB modulator BCL3 have been observed in lymphoid malignancies. The chromosomal translocation t(14;19)(q32;12) which juxtaposes *BCL3* with the *IGH* locus has been reported to result in an enhanced expression of BCL3 in a variety of lymphoid cancers, such as CLL and less commonly follicular lymphoma as well as marginal-zone lymphoma (Figure 6c) [226,227,228]. Additionally, amplification as well as alterations in the epigenetic modification status of the *BCL3* locus have been observed in HL and anaplastic large cell lymphomas [229,230,231]. The putative oncogenic role of BCL3 is supported by an *Eµ-BCL3* transgenic mouse model, which exhibits a lymphoproliferative disorder [232]. How exactly BCL3 exerts its oncogenic role in leukemia and lymphoma is unclear, but it has been proposed that BCL3 can promote cell proliferation and survival by transactivating a number of different target genes [233,234].

IκBζ (encoded by *NFKBIZ*) comprises a conserved NLS, a putative TAD as well as seven ankyrin repeats and is thus structurally highly homologous to the atypical IκB protein BCL3 [235,236]. Like BCL3, IκBζ interacts with NF-κB proteins, in particular with p50 and p52 homodimers, and is able to regulate the transcription of NF-κB target genes in a positive or negative manner [7]. Inhibition of target gene expression might be mediated by the stabilization of DNA-bound transcriptionally repressive p50 and p52 homodimers or by a competition between IκBζ and activating NF-κB members for DNA binding sites. Two molecular mechanisms have been proposed for its role as transcriptional activator: (I) Similar to BCL3, an intrinsic transactivation domain has been identified [237]. (II) IκBζ is capable to recruit the SWI/SNF complex, which mediates chromatin remodeling and thus allows transcription, to NF-κB consensus sites in the promoter of target genes [238].

In non-stimulated lymphocytes, IκBζ is not expressed but is rapidly induced upon engagement of receptors triggering NF-κB activity, such as TLRs and the antigen receptors [239,240]. Overexpression of IκBζ has been reported in various lymphoid malignancies, including ABC DLBCL, ATLL, and primary central nervous system lymphomas [241,242,243]. The expression of IκBζ is either promoted by genomic amplification of the *NFKBIZ* locus or by chronic NF-κB activation, which can be driven by deregulated BCR/IL-1R/TLR signaling or by viral proteins like Tax (HTLV-1) and LMP1 (EBV) (Figure 6d) [242,243,244]. The oncogenic function of IκBζ is best characterized in ABC DLBCL, since silencing of IκBζ reduced the growth of ABC DLBCL cell lines. Gene expression profiling revealed that IκBζ promotes the expression of several NF-κB target genes, including BCL-X_L_, IL-6, and IL-10, which represent key regulators for ABC DLBCL survival [242].

Collectively, modulation of the transcriptional activity of NF-κB in the nucleus by the atypical IκB proteins BCL3 and IκBζ potently affects the oncogenic potential of NF-κB in several lymphoid malignancies. As important regulators of cell proliferation and survival, BCL3 and IκBζ might emerge as attractive therapeutic targets to dampen excessive NF-κB activity in certain lymphoid cancers, possibly by pharmacologically preventing the interaction between p50 and BCL3 or IκBζ [245].

## 7. Conclusions and Implications for Lymphoma Therapy

To date, a large number of lymphoid malignancies has been found to harbor diverse genetic lesions that result in aberrant NF-κB activity. While some of these alterations are unique to specific lymphoma entities, other aberrations, such as inactivation of A20, commonly occur in a broad range of lymphoid tumors. Since several lymphoid cancers have been found to critically rely on constitutive NF-κB activity for their survival and since the anti-apoptotic effects of NF-κB activation can confer resistance towards cancer chemotherapy [246], inhibition of NF-κB activation represents an attractive therapeutic option in many lymphoid malignancies. As a master regulator of canonical NF-κB signaling, the IKK complex and in particular IKKβ, constitutes a promising therapeutic target. However, the crucial and pleiotropic role of NF-κB in many physiological processes is reflected, for instance, in the embryonic lethality associated with massive hepatocyte apoptosis in mice deficient for IKKγ and IKKβ, two major constituents of the IKK complex [247,248,249]. The expected systemic toxicity, immunosuppression and, paradoxically, increased IL-1β-mediated inflammation critically limit the therapeutic usefulness of general inhibition of canonical NF-κB, e.g., by pharmacological IKKβ inhibitors [250,251,252]. Thus, targeting of deregulated upstream pathways, such as chronic active BCR signaling, which drive constitutive NF-κB activation, potentially offers higher specificity for the malignant cells and represents an attractive alternative in the treatment of lymphoid malignancies. The validity of this concept has first been demonstrated by the therapeutic efficacy of the BTK inhibitor ibrutinib in ABC DLBCL and other lymphoid cancers relying on chronic BCR signaling [67,89,253,254]. Additional therapeutic targets in lymphoid tumors addicted to chronic BCR activation include SYK, LYN, and PKCβ [67,255,256]. The occurrence of primary resistance towards BTK inhibition due to oncogenic events targeting downstream effectors, such as CARMA1, as well as the acquisition of secondary resistance demonstrates the necessity of alternative therapeutic strategies and the rational stratification of patients regarding the mutational status of their lymphoid cancer [257]. While ABC DLBCL cells harboring CARMA1 mutations are insensitive towards inhibitors targeting upstream kinases involved in chronic BCR signaling, these cells still respond to treatment with inhibitors targeting MALT1 protease activity [256,258]. In addition to ABC DLBCL, MALT1 protease inhibition might also be beneficial for patients suffering from other lymphoid malignancies relying on constitutive CBM signaling or aberrant MALT1 protease activity, such as MCL and CLL as well as MALT lymphomas expressing the cIAP2-MALT1 fusion [150,151,152,153]. MALT1 protease inhibitors might prove especially valuable in the treatment of lymphomas that harbor mutations in signaling effectors downstream of BTK or that have acquired resistance towards BTK inhibition [98,136]. Besides aberrant activity of the CBM complex, constitutive NF-κB activation can also result from deregulation of the MyD88-IRAK4 signaling axis due to recurrent oncogenic MyD88 mutations. Recently, several small-molecule inhibitors selectively targeting IRAK4 have been shown to effectively abrogate aberrant NF-κB activation induced by MyD88^L265P^ in ABC DLBCL, thus representing an attractive therapeutic strategy for the treatment of MyD88 mutant lymphoid malignancies [40,50]. In addition to the genetic lesions promoting the constitutive activation of the canonical NF-κB pathway, several lymphoid cancers rely on the aberrant activity of non-canonical NF-κB signaling. The majority of these genetic aberrations result in the stabilization or increased expression of the central kinase NIK, the activity of which is essential for the survival of these lymphomas [19,20]. Even though no NIK inhibitor has entered the clinic yet, NIK represents a promising therapeutic target that should be addressed more vigorously, particularly regarding the treatment of multiple myeloma and MALT lymphoma expressing the oncogenic cIAP2-MALT1 fusion protein [188,259].

In light of the variety of genetic lesions in lymphoid malignancies which can cause constitutive NF-κB activation through deregulation of distinct upstream signaling nodes, the ultimate goal in the treatment of cancer, i.e., the highly specific eradication of the tumor cells by a targeted therapy, requires the analysis of the relevant oncogenic lesions and deregulated signaling pathways in the respective lymphoid tumor. The knowledge about how a certain oncogenic lesion drives NF-κB activation as well as the identification and molecular characterization of novel oncogenic mechanisms governing lymphomagenesis will pave the way for the rational design of therapeutic strategies, for instance by simultaneously targeting complementary signaling pathways, and thus improve lymphoma therapy.

## References

[1] Liu F., Xia Y., Parker A.S., Verma I.M. (2012). IKK biology. Immunol. Rev..

[2] Ghosh S., Hayden M.S. (2008). New regulators of NF-κB in inflammation. Nat. Rev. Immunol..

[3] Oeckinghaus A., Ghosh S. (2009). The NF-κB family of transcription factors and its regulation. Cold Spring Harb. Perspect. Biol..

[4] Sun S.C. (2012). The noncanonical NF-κB pathway. Immunol. Rev..

[5] Schuster M., Annemann M., Plaza-Sirvent C., Schmitz I. (2013). Atypical IκB proteins–nuclear modulators of NF-κB signaling. Cell Commun. Signal..

[6] Hinz M., Arslan S.C., Scheidereit C. (2012). It takes two to tango: IκBs, the multifunctional partners of NF-κB. Immunol. Rev..

[7] Annemann M., Plaza-Sirvent C., Schuster M., Katsoulis-Dimitriou K., Kliche S., Schraven B., Schmitz I. (2016). Atypical IκB proteins in immune cell differentiation and function. Immunol. Lett..

[8] Kaileh M., Sen R. (2012). NF-κB function in B lymphocytes. Immunol. Rev..

[9] Staudt L.M. (2010). Oncogenic activation of NF-κB. Cold Spring Harb. Perspect. Biol..

[10] Lim K.H., Yang Y., Staudt L.M. (2012). Pathogenetic importance and therapeutic implications of NF-κB in lymphoid malignancies. Immunol. Rev..

[11] Beug H., Muller H., Grieser S., Doederlein G., Graf T. (1981). Hematopoietic cells transformed in vitro by REVT avian reticuloendotheliosis virus express characteristics of very immature lymphoid cells. Virology.

[12] Barth C.F., Ewert D.L., Olson W.C., Humphries E.H. (1990). Reticuloendotheliosis virus REV-T(REV-A)-induced neoplasia: Development of tumors within the T-lymphoid and myeloid lineages. J. Virol..

[13] Lu D., Thompson J.D., Gorski G.K., Rice N.R., Mayer M.G., Yunis J.J. (1991). Alterations at the rel locus in human lymphoma. Oncogene.

[14] Gilmore T.D., Gerondakis S. (2011). The c-Rel Transcription Factor in Development and Disease. Genes Cancer.

[15] Bargou R.C., Leng C., Krappmann D., Emmerich F., Mapara M.Y., Bommert K., Royer H.D., Scheidereit C., Dorken B. (1996). High-level nuclear NF-κB and Oct-2 is a common feature of cultured Hodgkin/Reed-Sternberg cells. Blood.

[16] Davis R.E., Brown K.D., Siebenlist U., Staudt L.M. (2001). Constitutive nuclear factor κB activity is required for survival of activated B cell-like diffuse large B cell lymphoma cells. J. Exp. Med..

[17] Ni H., Ergin M., Huang Q., Qin J.Z., Amin H.M., Martinez R.L., Saeed S., Barton K., Alkan S. (2001). Analysis of expression of nuclear factor kappa B (NF-κB) in multiple myeloma: Downregulation of NF-κB induces apoptosis. Br. J. Haematol..

[18] Savage K.J., Monti S., Kutok J.L., Cattoretti G., Neuberg D., De Leval L., Kurtin P., Dal Cin P., Ladd C., Feuerhake F. (2003). The molecular signature of mediastinal large B-cell lymphoma differs from that of other diffuse large B-cell lymphomas and shares features with classical Hodgkin lymphoma. Blood.

[19] Keats J.J., Fonseca R., Chesi M., Schop R., Baker A., Chng W.J., Van Wier S., Tiedemann R., Shi C.X., Sebag M. (2007). Promiscuous mutations activate the noncanonical NF-κB pathway in multiple myeloma. Cancer Cell.

[20] Annunziata C.M., Davis R.E., Demchenko Y., Bellamy W., Gabrea A., Zhan F., Lenz G., Hanamura I., Wright G., Xiao W. (2007). Frequent engagement of the classical and alternative NF-κB pathways by diverse genetic abnormalities in multiple myeloma. Cancer Cell.

[21] Shaffer A.L., Young R.M., Staudt L.M. (2012). Pathogenesis of human B cell lymphomas. Annu. Rev. Immunol..

[22] Herishanu Y., Perez-Galan P., Liu D., Biancotto A., Pittaluga S., Vire B., Gibellini F., Njuguna N., Lee E., Stennett L. (2011). The lymph node microenvironment promotes B-cell receptor signaling, NF-κB activation, and tumor proliferation in chronic lymphocytic leukemia. Blood.

[23] Rahal R., Frick M., Romero R., Korn J.M., Kridel R., Chan F.C., Meissner B., Bhang H.E., Ruddy D., Kauffmann A. (2014). Pharmacological and genomic profiling identifies NF-κB-targeted treatment strategies for mantle cell lymphoma. Nat. Med..

[24] Rosebeck S., Madden L., Jin X., Gu S., Apel I.J., Appert A., Hamoudi R.A., Noels H., Sagaert X., Van Loo P. (2011). Cleavage of NIK by the API2-MALT1 fusion oncoprotein leads to noncanonical NF-κB activation. Science.

[25] Young R.M., Shaffer A.L., Phelan J.D., Staudt L.M. (2015). B-cell receptor signaling in diffuse large B-cell lymphoma. Semin. Hematol..

[26] Takeda K., Akira S. (2004). Microbial recognition by Toll-like receptors. J. Dermatol. Sci..

[27] Medzhitov R., Preston-Hurlburt P., Kopp E., Stadlen A., Chen C., Ghosh S., Janeway C.A. (1998). MyD88 is an adaptor protein in the hToll/IL-1 receptor family signaling pathways. Mol. Cell.

[28] Lin S.C., Lo Y.C., Wu H. (2010). Helical assembly in the MyD88-IRAK4-IRAK2 complex in TLR/IL-1R signalling. Nature.

[29] Wesche H., Henzel W.J., Shillinglaw W., Li S., Cao Z. (1997). MyD88: An adapter that recruits IRAK to the IL-1 receptor complex. Immunity.

[30] Muzio M., Ni J., Feng P., Dixit V.M. (1997). IRAK (Pelle) family member IRAK-2 and MyD88 as proximal mediators of IL-1 signaling. Science.

[31] Vollmer S., Strickson S., Zhang T., Gray N., Lee K.L., Rao V.R., Cohen P. (2017). The mechanism of activation of IRAK1 and IRAK4 by interleukin-1 and Toll-like receptor agonists. Biochem. J..

[32] Cheng H., Addona T., Keshishian H., Dahlstrand E., Lu C., Dorsch M., Li Z., Wang A., Ocain T.D., Li P. (2007). Regulation of IRAK-4 kinase activity via autophosphorylation within its activation loop. Biochem. Biophys. Res. Commun..

[33] Kollewe C., Mackensen A.C., Neumann D., Knop J., Cao P., Li S., Wesche H., Martin M.U. (2004). Sequential autophosphorylation steps in the interleukin-1 receptor-associated kinase-1 regulate its availability as an adapter in interleukin-1 signaling. J. Biol. Chem..

[34] Dossang A.C., Motshwene P.G., Yang Y., Symmons M.F., Bryant C.E., Borman S., George J., Weber A.N., Gay N.J. (2016). The N-terminal loop of IRAK-4 death domain regulates ordered assembly of the Myddosome signalling scaffold. Sci. Rep..

[35] Keating S.E., Maloney G.M., Moran E.M., Bowie A.G. (2007). IRAK-2 participates in multiple toll-like receptor signaling pathways to NFκB via activation of TRAF6 ubiquitination. J. Biol. Chem..

[36] Ye H., Arron J.R., Lamothe B., Cirilli M., Kobayashi T., Shevde N.K., Segal D., Dzivenu O.K., Vologodskaia M., Yim M. (2002). Distinct molecular mechanism for initiating TRAF6 signalling. Nature.

[37] Deng L., Wang C., Spencer E., Yang L., Braun A., You J., Slaughter C., Pickart C., Chen Z.J. (2000). Activation of the IκB kinase complex by TRAF6 requires a dimeric ubiquitin-conjugating enzyme complex and a unique polyubiquitin chain. Cell.

[38] Takaesu G., Kishida S., Hiyama A., Yamaguchi K., Shibuya H., Irie K., Ninomiya-Tsuji J., Matsumoto K. (2000). TAB2, a novel adaptor protein, mediates activation of TAK1 MAPKKK by linking TAK1 to TRAF6 in the IL-1 signal transduction pathway. Mol. Cell.

[39] Wang C., Deng L., Hong M., Akkaraju G.R., Inoue J., Chen Z.J. (2001). TAK1 is a ubiquitin-dependent kinase of MKK and IKK. Nature.

[40] Ngo V.N., Young R.M., Schmitz R., Jhavar S., Xiao W., Lim K.H., Kohlhammer H., Xu W., Yang Y., Zhao H. (2011). Oncogenically active MYD88 mutations in human lymphoma. Nature.

[41] Puente X.S., Pinyol M., Quesada V., Conde L., Ordonez G.R., Villamor N., Escaramis G., Jares P., Bea S., Gonzalez-Diaz M. (2011). Whole-genome sequencing identifies recurrent mutations in chronic lymphocytic leukaemia. Nature.

[42] Yan Q., Huang Y., Watkins A.J., Kocialkowski S., Zeng N., Hamoudi R.A., Isaacson P.G., de Leval L., Wotherspoon A., Du M.Q. (2012). BCR and TLR signaling pathways are recurrently targeted by genetic changes in splenic marginal zone lymphomas. Haematologica.

[43] Treon S.P., Xu L., Yang G., Zhou Y., Liu X., Cao Y., Sheehy P., Manning R.J., Patterson C.J., Tripsas C. (2012). MYD88 L265P somatic mutation in Waldenstrom’s macroglobulinemia. N. Engl. J. Med..

[44] Troen G., Warsame A., Delabie J. (2013). CD79B and MYD88 Mutations in Splenic Marginal Zone Lymphoma. ISRN Oncol..

[45] Pasqualucci L., Trifonov V., Fabbri G., Ma J., Rossi D., Chiarenza A., Wells V.A., Grunn A., Messina M., Elliot O. (2011). Analysis of the coding genome of diffuse large B-cell lymphoma. Nat. Genet..

[46] Avbelj M., Wolz O.O., Fekonja O., Bencina M., Repic M., Mavri J., Kruger J., Scharfe C., Delmiro Garcia M., Panter G. (2014). Activation of lymphoma-associated MyD88 mutations via allostery-induced TIR-domain oligomerization. Blood.

[47] Knittel G., Liedgens P., Korovkina D., Seeger J.M., Al-Baldawi Y., Al-Maarri M., Fritz C., Vlantis K., Bezhanova S., Scheel A.H. (2016). B-cell-specific conditional expression of Myd88p.L252P leads to the development of diffuse large B-cell lymphoma in mice. Blood.

[48] Zhan C., Qi R., Wei G., Guven-Maiorov E., Nussinov R., Ma B. (2016). Conformational dynamics of cancer-associated MyD88-TIR domain mutant L252P (L265P) allosterically tilts the landscape toward homo-dimerization. Protein Eng. Des. Sel..

[49] Loiarro M., Volpe E., Ruggiero V., Gallo G., Furlan R., Maiorino C., Battistini L., Sette C. (2013). Mutational analysis identifies residues crucial for homodimerization of myeloid differentiation factor 88 (MyD88) and for its function in immune cells. J. Biol. Chem..

[50] Kelly P.N., Romero D.L., Yang Y., Shaffer A.L., Chaudhary D., Robinson S., Miao W., Rui L., Westlin W.F., Kapeller R. (2015). Selective interleukin-1 receptor-associated kinase 4 inhibitors for the treatment of autoimmune disorders and lymphoid malignancy. J. Exp. Med..

[51] Yang G., Zhou Y., Liu X., Xu L., Cao Y., Manning R.J., Patterson C.J., Buhrlage S.J., Gray N., Tai Y.T. (2013). A mutation in MYD88 (L265P) supports the survival of lymphoplasmacytic cells by activation of Bruton tyrosine kinase in Waldenstrom macroglobulinemia. Blood.

[52] Wang J.Q., Jeelall Y.S., Beutler B., Horikawa K., Goodnow C.C. (2014). Consequences of the recurrent MYD88(L265P) somatic mutation for B cell tolerance. J. Exp. Med..

[53] Wang J.Q., Beutler B., Goodnow C.C., Horikawa K. (2016). Inhibiting TLR9 and other UNC93B1-dependent TLRs paradoxically increases accumulation of MYD88L265P plasmablasts in vivo. Blood.

[54] Reth M. (1992). Antigen receptors on B lymphocytes. Annu. Rev. Immunol..

[55] Wienands J., Engels N. (2001). Multitasking of Ig-α and Ig-β to regulate B cell antigen receptor function. Int. Rev. Immunol..

[56] Johnson S.A., Pleiman C.M., Pao L., Schneringer J., Hippen K., Cambier J.C. (1995). Phosphorylated immunoreceptor signaling motifs (ITAMs) exhibit unique abilities to bind and activate Lyn and Syk tyrosine kinases. J. Immunol..

[57] Harwood N.E., Batista F.D. (2010). Early events in B cell activation. Annu. Rev. Immunol..

[58] Kurosaki T., Hikida M. (2009). Tyrosine kinases and their substrates in B lymphocytes. Immunol. Rev..

[59] Fu C., Turck C.W., Kurosaki T., Chan A.C. (1998). BLNK: A central linker protein in B cell activation. Immunity.

[60] Watanabe D., Hashimoto S., Ishiai M., Matsushita M., Baba Y., Kishimoto T., Kurosaki T., Tsukada S. (2001). Four tyrosine residues in phospholipase C-γ2, identified as Btk-dependent phosphorylation sites, are required for B cell antigen receptor-coupled calcium signaling. J. Biol. Chem..

[61] Thome M., Charton J.E., Pelzer C., Hailfinger S. (2010). Antigen receptor signaling to NF-κB via CARMA1, BCL10, and MALT1. Cold Spring Harb. Perspect. Biol..

[62] Knittel G., Liedgens P., Korovkina D., Pallasch C.P., Reinhardt H.C. (2016). Rewired NFκB signaling as a potentially actionable feature of activated B-cell-like diffuse large B-cell lymphoma. Eur. J. Haematol..

[63] Suarez F., Lortholary O., Hermine O., Lecuit M. (2006). Infection-associated lymphomas derived from marginal zone B cells: A model of antigen-driven lymphoproliferation. Blood.

[64] Quinn E.R., Chan C.H., Hadlock K.G., Foung S.K., Flint M., Levy S. (2001). The B-cell receptor of a hepatitis C virus (HCV)-associated non-Hodgkin lymphoma binds the viral E2 envelope protein, implicating HCV in lymphomagenesis. Blood.

[65] Young R.M., Wu T., Schmitz R., Dawood M., Xiao W., Phelan J.D., Xu W., Menard L., Meffre E., Chan W.C. (2015). Survival of human lymphoma cells requires B-cell receptor engagement by self-antigens. Proc. Natl. Acad. Sci. USA.

[66] Ngo V.N., Davis R.E., Lamy L., Yu X., Zhao H., Lenz G., Lam L.T., Dave S., Yang L., Powell J. (2006). A loss-of-function RNA interference screen for molecular targets in cancer. Nature.

[67] Davis R.E., Ngo V.N., Lenz G., Tolar P., Young R.M., Romesser P.B., Kohlhammer H., Lamy L., Zhao H., Yang Y. (2010). Chronic active B-cell-receptor signalling in diffuse large B-cell lymphoma. Nature.

[68] Havranek O., Xu J., Kohrer S., Wang Z., Becker L., Comer J.M., Henderson J., Ma W., Man Chun Ma J., Westin J.R. (2017). Tonic B-cell receptor signaling in diffuse large B-cell lymphoma. Blood.

[69] Srinivasan L., Sasaki Y., Calado D.P., Zhang B., Paik J.H., DePinho R.A., Kutok J.L., Kearney J.F., Otipoby K.L., Rajewsky K. (2009). PI3 kinase signals BCR-dependent mature B cell survival. Cell.

[70] Kraus M., Alimzhanov M.B., Rajewsky N., Rajewsky K. (2004). Survival of resting mature B lymphocytes depends on BCR signaling via the Igα/β heterodimer. Cell.

[71] Agathangelidis A., Darzentas N., Hadzidimitriou A., Brochet X., Murray F., Yan X.J., Davis Z., van Gastel-Mol E.J., Tresoldi C., Chu C.C. (2012). Stereotyped B-cell receptors in one-third of chronic lymphocytic leukemia: A molecular classification with implications for targeted therapies. Blood.

[72] Catera R., Silverman G.J., Hatzi K., Seiler T., Didier S., Zhang L., Herve M., Meffre E., Oscier D.G., Vlassara H. (2008). Chronic lymphocytic leukemia cells recognize conserved epitopes associated with apoptosis and oxidation. Mol. Med..

[73] Chu C.C., Catera R., Zhang L., Didier S., Agagnina B.M., Damle R.N., Kaufman M.S., Kolitz J.E., Allen S.L., Rai K.R. (2010). Many chronic lymphocytic leukemia antibodies recognize apoptotic cells with exposed nonmuscle myosin heavy chain IIA: Implications for patient outcome and cell of origin. Blood.

[74] Duhren-von Minden M., Ubelhart R., Schneider D., Wossning T., Bach M.P., Buchner M., Hofmann D., Surova E., Follo M., Kohler F. (2012). Chronic lymphocytic leukaemia is driven by antigen-independent cell-autonomous signalling. Nature.

[75] Gazumyan A., Reichlin A., Nussenzweig M.C. (2006). Igβ tyrosine residues contribute to the control of B cell receptor signaling by regulating receptor internalization. J. Exp. Med..

[76] Chan V.W., Lowell C.A., DeFranco A.L. (1998). Defective negative regulation of antigen receptor signaling in Lyn-deficient B lymphocytes. Curr. Biol..

[77] Doody G.M., Justement L.B., Delibrias C.C., Matthews R.J., Lin J., Thomas M.L., Fearon D.T. (1995). A role in B cell activation for CD22 and the protein tyrosine phosphatase SHP. Science.

[78] Nishizumi H., Horikawa K., Mlinaric-Rascan I., Yamamoto T. (1998). A double-edged kinase Lyn: A positive and negative regulator for antigen receptor-mediated signals. J. Exp. Med..

[79] Chan V.W., Meng F., Soriano P., DeFranco A.L., Lowell C.A. (1997). Characterization of the B lymphocyte populations in Lyn-deficient mice and the role of Lyn in signal initiation and down-regulation. Immunity.

[80] Cornall R.J., Cyster J.G., Hibbs M.L., Dunn A.R., Otipoby K.L., Clark E.A., Goodnow C.C. (1998). Polygenic autoimmune traits: Lyn, CD22, and SHP-1 are limiting elements of a biochemical pathway regulating BCR signaling and selection. Immunity.

[81] Wang J.Q., Jeelall Y.S., Humburg P., Batchelor E.L., Kaya S.M., Yoo H.M., Goodnow C.C., Horikawa K. (2017). Synergistic cooperation and crosstalk between MYD88(L265P) and mutations that dysregulate CD79B and surface IgM. J. Exp. Med..

[82] Hunter Z.R., Xu L., Yang G., Zhou Y., Liu X., Cao Y., Manning R.J., Tripsas C., Patterson C.J., Sheehy P. (2014). The genomic landscape of Waldenstrom macroglobulinemia is characterized by highly recurring MYD88 and WHIM-like CXCR4 mutations, and small somatic deletions associated with B-cell lymphomagenesis. Blood.

[83] Rinaldi A., Kwee I., Taborelli M., Largo C., Uccella S., Martin V., Poretti G., Gaidano G., Calabrese G., Martinelli G. (2006). Genomic and expression profiling identifies the B-cell associated tyrosine kinase Syk as a possible therapeutic target in mantle cell lymphoma. Br. J. Haematol..

[84] Feldman A.L., Sun D.X., Law M.E., Novak A.J., Attygalle A.D., Thorland E.C., Fink S.R., Vrana J.A., Caron B.L., Morice W.G. (2008). Overexpression of Syk tyrosine kinase in peripheral T-cell lymphomas. Leukemia.

[85] Burger J.A., Wiestner A. (2018). Targeting B cell receptor signalling in cancer: Preclinical and clinical advances. Nat. Rev. Cancer.

[86] Burger J.A., Tedeschi A., Barr P.M., Robak T., Owen C., Ghia P., Bairey O., Hillmen P., Bartlett N.L., Li J. (2015). Ibrutinib as Initial Therapy for Patients with Chronic Lymphocytic Leukemia. N. Engl. J. Med..

[87] Kim E.S., Dhillon S. (2015). Ibrutinib: A review of its use in patients with mantle cell lymphoma or chronic lymphocytic leukaemia. Drugs.

[88] Treon S.P., Tripsas C.K., Meid K., Warren D., Varma G., Green R., Argyropoulos K.V., Yang G., Cao Y., Xu L. (2015). Ibrutinib in previously treated Waldenstrom’s macroglobulinemia. N. Engl. J. Med..

[89] Wilson W.H., Young R.M., Schmitz R., Yang Y., Pittaluga S., Wright G., Lih C.J., Williams P.M., Shaffer A.L., Gerecitano J. (2015). Targeting B cell receptor signaling with ibrutinib in diffuse large B cell lymphoma. Nat. Med..

[90] Byrd J.C., Harrington B., O’Brien S., Jones J.A., Schuh A., Devereux S., Chaves J., Wierda W.G., Awan F.T., Brown J.R. (2016). Acalabrutinib (ACP-196) in Relapsed Chronic Lymphocytic Leukemia. N. Engl. J. Med..

[91] Kuiatse I., Baladandayuthapani V., Lin H.Y., Thomas S.K., Bjorklund C.C., Weber D.M., Wang M., Shah J.J., Zhang X.D., Jones R.J. (2015). Targeting the Spleen Tyrosine Kinase with Fostamatinib as a Strategy against Waldenstrom Macroglobulinemia. Clin. Cancer Res..

[92] Flinn I.W., Bartlett N.L., Blum K.A., Ardeshna K.M., LaCasce A.S., Flowers C.R., Shustov A.R., Thress K.S., Mitchell P., Zheng F. (2016). A phase II trial to evaluate the efficacy of fostamatinib in patients with relapsed or refractory diffuse large B-cell lymphoma (DLBCL). Eur. J. Cancer.

[93] Hantschel O., Rix U., Schmidt U., Burckstummer T., Kneidinger M., Schutze G., Colinge J., Bennett K.L., Ellmeier W., Valent P. (2007). The Btk tyrosine kinase is a major target of the Bcr-Abl inhibitor dasatinib. Proc. Natl. Acad. Sci. USA.

[94] Amrein P.C., Attar E.C., Takvorian T., Hochberg E.P., Ballen K.K., Leahy K.M., Fisher D.C., Lacasce A.S., Jacobsen E.D., Armand P. (2011). Phase II study of dasatinib in relapsed or refractory chronic lymphocytic leukemia. Clin. Cancer Res..

[95] Lindauer M., Hochhaus A. (2014). Dasatinib. Recent Results Cancer Res..

[96] Ruland J., Duncan G.S., Wakeham A., Mak T.W. (2003). Differential requirement for Malt1 in T and B cell antigen receptor signaling. Immunity.

[97] Thome M. (2004). CARMA1, BCL-10 and MALT1 in lymphocyte development and activation. Nat. Rev. Immunol..

[98] Juilland M., Thome M. (2016). Role of the CARMA1/BCL10/MALT1 complex in lymphoid malignancies. Curr. Opin. Hematol..

[99] Matsumoto R., Wang D., Blonska M., Li H., Kobayashi M., Pappu B., Chen Y., Wang D., Lin X. (2005). Phosphorylation of CARMA1 plays a critical role in T Cell receptor-mediated NF-κB activation. Immunity.

[100] Sommer K., Guo B., Pomerantz J.L., Bandaranayake A.D., Moreno-Garcia M.E., Ovechkina Y.L., Rawlings D.J. (2005). Phosphorylation of the CARMA1 linker controls NF-κB activation. Immunity.

[101] Tanner M.J., Hanel W., Gaffen S.L., Lin X. (2007). CARMA1 coiled-coil domain is involved in the oligomerization and subcellular localization of CARMA1 and is required for T cell receptor-induced NF-κB activation. J. Biol. Chem..

[102] Blonska M., Lin X. (2011). NF-κB signaling pathways regulated by CARMA family of scaffold proteins. Cell Res..

[103] Qiao Q., Yang C., Zheng C., Fontan L., David L., Yu X., Bracken C., Rosen M., Melnick A., Egelman E.H. (2013). Structural architecture of the CARMA1/Bcl10/MALT1 signalosome: Nucleation-induced filamentous assembly. Mol. Cell.

[104] David L., Li Y., Ma J., Garner E., Zhang X., Wu H. (2018). Assembly mechanism of the CARMA1-BCL10-MALT1-TRAF6 signalosome. Proc. Natl. Acad. Sci. USA.

[105] Bertin J., Wang L., Guo Y., Jacobson M.D., Poyet J.L., Srinivasula S.M., Merriam S., DiStefano P.S., Alnemri E.S. (2001). CARD11 and CARD14 are novel caspase recruitment domain (CARD)/membrane-associated guanylate kinase (MAGUK) family members that interact with BCL10 and activate NF-κB. J. Biol. Chem..

[106] Gaide O., Martinon F., Micheau O., Bonnet D., Thome M., Tschopp J. (2001). Carma1, a CARD-containing binding partner of Bcl10, induces Bcl10 phosphorylation and NF-κB activation. FEBS Lett..

[107] Uren A.G., O’Rourke K., Aravind L.A., Pisabarro M.T., Seshagiri S., Koonin E.V., Dixit V.M. (2000). Identification of paracaspases and metacaspases: Two ancient families of caspase-like proteins, one of which plays a key role in MALT lymphoma. Mol. Cell.

[108] Lucas P.C., Yonezumi M., Inohara N., McAllister-Lucas L.M., Abazeed M.E., Chen F.F., Yamaoka S., Seto M., Nunez G. (2001). Bcl10 and MALT1, independent targets of chromosomal translocation in malt lymphoma, cooperate in a novel NF-κB signaling pathway. J. Biol. Chem..

[109] McAllister-Lucas L.M., Inohara N., Lucas P.C., Ruland J., Benito A., Li Q., Chen S., Chen F.F., Yamaoka S., Verma I.M. (2001). Bimp1, a MAGUK family member linking protein kinase C activation to Bcl10-mediated NF-κB induction. J. Biol. Chem..

[110] Jaworski M., Thome M. (2016). The paracaspase MALT1: Biological function and potential for therapeutic inhibition. Cell. Mol. Life Sci..

[111] Oeckinghaus A., Wegener E., Welteke V., Ferch U., Arslan S.C., Ruland J., Scheidereit C., Krappmann D. (2007). Malt1 ubiquitination triggers NF-κB signaling upon T-cell activation. EMBO J..

[112] Sun L., Deng L., Ea C.K., Xia Z.P., Chen Z.J. (2004). The TRAF6 ubiquitin ligase and TAK1 kinase mediate IKK activation by BCL10 and MALT1 in T lymphocytes. Mol. Cell.

[113] Wu C.J., Ashwell J.D. (2008). NEMO recognition of ubiquitinated Bcl10 is required for T cell receptor-mediated NF-κB activation. Proc. Natl. Acad. Sci. USA.

[114] Thome M. (2008). Multifunctional roles for MALT1 in T-cell activation. Nat. Rev. Immunol..

[115] Fujita H., Rahighi S., Akita M., Kato R., Sasaki Y., Wakatsuki S., Iwai K. (2014). Mechanism underlying IκB kinase activation mediated by the linear ubiquitin chain assembly complex. Mol. Cell Biol..

[116] Tokunaga F., Sakata S., Saeki Y., Satomi Y., Kirisako T., Kamei K., Nakagawa T., Kato M., Murata S., Yamaoka S. (2009). Involvement of linear polyubiquitylation of NEMO in NF-κB activation. Nat. Cell Biol..

[117] Rahighi S., Ikeda F., Kawasaki M., Akutsu M., Suzuki N., Kato R., Kensche T., Uejima T., Bloor S., Komander D. (2009). Specific recognition of linear ubiquitin chains by NEMO is important for NF-κB activation. Cell.

[118] Adhikari A., Xu M., Chen Z.J. (2007). Ubiquitin-mediated activation of TAK1 and IKK. Oncogene.

[119] Vercammen D., Declercq W., Vandenabeele P., Van Breusegem F. (2007). Are metacaspases caspases?. J. Cell Biol..

[120] Rebeaud F., Hailfinger S., Posevitz-Fejfar A., Tapernoux M., Moser R., Rueda D., Gaide O., Guzzardi M., Iancu E.M., Rufer N. (2008). The proteolytic activity of the paracaspase MALT1 is key in T cell activation. Nat. Immunol..

[121] Coornaert B., Baens M., Heyninck K., Bekaert T., Haegman M., Staal J., Sun L., Chen Z.J., Marynen P., Beyaert R. (2008). T cell antigen receptor stimulation induces MALT1 paracaspase-mediated cleavage of the NF-κB inhibitor A20. Nat. Immunol..

[122] Cabalzar K., Pelzer C., Wolf A., Lenz G., Iwaszkiewicz J., Zoete V., Hailfinger S., Thome M. (2013). Monoubiquitination and activity of the paracaspase MALT1 requires glutamate 549 in the dimerization interface. PLoS ONE.

[123] Pelzer C., Cabalzar K., Wolf A., Gonzalez M., Lenz G., Thome M. (2013). The protease activity of the paracaspase MALT1 is controlled by monoubiquitination. Nat. Immunol..

[124] Wiesmann C., Leder L., Blank J., Bernardi A., Melkko S., Decock A., D’Arcy A., Villard F., Erbel P., Hughes N. (2012). Structural determinants of MALT1 protease activity. J. Mol. Biol..

[125] Hailfinger S., Nogai H., Pelzer C., Jaworski M., Cabalzar K., Charton J.E., Guzzardi M., Decaillet C., Grau M., Dorken B. (2011). Malt1-dependent RelB cleavage promotes canonical NF-κB activation in lymphocytes and lymphoma cell lines. Proc. Natl. Acad. Sci. USA.

[126] Marienfeld R., May M.J., Berberich I., Serfling E., Ghosh S., Neumann M. (2003). RelB forms transcriptionally inactive complexes with RelA/p65. J. Biol. Chem..

[127] Duwel M., Welteke V., Oeckinghaus A., Baens M., Kloo B., Ferch U., Darnay B.G., Ruland J., Marynen P., Krappmann D. (2009). A20 negatively regulates T cell receptor signaling to NF-κB by cleaving Malt1 ubiquitin chains. J. Immunol..

[128] Baens M., Bonsignore L., Somers R., Vanderheydt C., Weeks S.D., Gunnarsson J., Nilsson E., Roth R.G., Thome M., Marynen P. (2014). MALT1 auto-proteolysis is essential for NF-κB-dependent gene transcription in activated lymphocytes. PLoS ONE.

[129] Klein T., Fung S.Y., Renner F., Blank M.A., Dufour A., Kang S., Bolger-Munro M., Scurll J.M., Priatel J.J., Schweigler P. (2015). The paracaspase MALT1 cleaves HOIL1 reducing linear ubiquitination by LUBAC to dampen lymphocyte NF-κB signalling. Nat. Commun..

[130] Elton L., Carpentier I., Staal J., Driege Y., Haegman M., Beyaert R. (2016). MALT1 cleaves the E3 ubiquitin ligase HOIL-1 in activated T cells, generating a dominant negative inhibitor of LUBAC-induced NF-κB signaling. FEBS J..

[131] Hailfinger S., Schmitt A., Schulze-Osthoff K. (2016). The paracaspase MALT1 dampens NF-κB signalling by cleaving the LUBAC subunit HOIL-1. FEBS J..

[132] Karin M., Cao Y., Greten F.R., Li Z.W. (2002). NF-κB in cancer: From innocent bystander to major culprit. Nat. Rev. Cancer.

[133] Li Q., Verma I.M. (2002). NF-κB regulation in the immune system. Nat. Rev. Immunol..

[134] Rosebeck S., Rehman A.O., Lucas P.C., McAllister-Lucas L.M. (2011). From MALT lymphoma to the CBM signalosome: Three decades of discovery. Cell Cycle.

[135] Da Silva Almeida A.C., Abate F., Khiabanian H., Martinez-Escala E., Guitart J., Tensen C.P., Vermeer M.H., Rabadan R., Ferrando A., Palomero T. (2015). The mutational landscape of cutaneous T cell lymphoma and Sezary syndrome. Nat. Genet..

[136] Young R.M., Staudt L.M. (2013). Targeting pathological B cell receptor signalling in lymphoid malignancies. Nat. Rev. Drug Discov..

[137] Lenz G., Davis R.E., Ngo V.N., Lam L., George T.C., Wright G.W., Dave S.S., Zhao H., Xu W., Rosenwald A. (2008). Oncogenic CARD11 mutations in human diffuse large B cell lymphoma. Science.

[138] Brohl A.S., Stinson J.R., Su H.C., Badgett T., Jennings C.D., Sukumar G., Sindiri S., Wang W., Kardava L., Moir S. (2015). Germline CARD11 Mutation in a Patient with Severe Congenital B Cell Lymphocytosis. J. Clin. Immunol..

[139] Snow A.L., Xiao W., Stinson J.R., Lu W., Chaigne-Delalande B., Zheng L., Pittaluga S., Matthews H.F., Schmitz R., Jhavar S. (2012). Congenital B cell lymphocytosis explained by novel germline CARD11 mutations. J. Exp. Med..

[140] Wang L., Ni X., Covington K.R., Yang B.Y., Shiu J., Zhang X., Xi L., Meng Q., Langridge T., Drummond J. (2015). Genomic profiling of Sezary syndrome identifies alterations of key T cell signaling and differentiation genes. Nat. Genet..

[141] Isaacson P.G., Du M.Q. (2004). MALT lymphoma: From morphology to molecules. Nat. Rev. Cancer.

[142] Wotherspoon A.C., Doglioni C., Diss T.C., Pan L., Moschini A., de Boni M., Isaacson P.G. (1993). Regression of primary low-grade B-cell gastric lymphoma of mucosa-associated lymphoid tissue type after eradication of Helicobacter pylori. Lancet.

[143] Streubel B., Lamprecht A., Dierlamm J., Cerroni L., Stolte M., Ott G., Raderer M., Chott A. (2003). T(14;18)(q32;q21) involving IGH and MALT1 is a frequent chromosomal aberration in MALT lymphoma. Blood.

[144] Willis T.G., Jadayel D.M., Du M.Q., Peng H., Perry A.R., Abdul-Rauf M., Price H., Karran L., Majekodunmi O., Wlodarska I. (1999). Bcl10 is involved in t(1;14)(p22;q32) of MALT B cell lymphoma and mutated in multiple tumor types. Cell.

[145] Zhang Q., Siebert R., Yan M., Hinzmann B., Cui X., Xue L., Rakestraw K.M., Naeve C.W., Beckmann G., Weisenburger D.D. (1999). Inactivating mutations and overexpression of BCL10, a caspase recruitment domain-containing gene, in MALT lymphoma with t(1;14)(p22;q32). Nat. Genet..

[146] Dierlamm J., Baens M., Wlodarska I., Stefanova-Ouzounova M., Hernandez J.M., Hossfeld D.K., De Wolf-Peeters C., Hagemeijer A., Van den Berghe H., Marynen P. (1999). The apoptosis inhibitor gene API2 and a novel 18q gene, MLT, are recurrently rearranged in the t(11;18)(q21;q21) associated with mucosa-associated lymphoid tissue lymphomas. Blood.

[147] Akagi T., Motegi M., Tamura A., Suzuki R., Hosokawa Y., Suzuki H., Ota H., Nakamura S., Morishima Y., Taniwaki M. (1999). A novel gene, MALT1 at 18q21, is involved in t(11;18) (q21;q21) found in low-grade B-cell lymphoma of mucosa-associated lymphoid tissue. Oncogene.

[148] Lucas P.C., Kuffa P., Gu S., Kohrt D., Kim D.S., Siu K., Jin X., Swenson J., McAllister-Lucas L.M. (2007). A dual role for the API2 moiety in API2-MALT1-dependent NF-κB activation: Heterotypic oligomerization and TRAF2 recruitment. Oncogene.

[149] Noels H., van Loo G., Hagens S., Broeckx V., Beyaert R., Marynen P., Baens M. (2007). A Novel TRAF6 binding site in MALT1 defines distinct mechanisms of NF-κB activation by API2middle dotMALT1 fusions. J. Biol. Chem..

[150] Ferch U., Kloo B., Gewies A., Pfander V., Duwel M., Peschel C., Krappmann D., Ruland J. (2009). Inhibition of MALT1 protease activity is selectively toxic for activated B cell-like diffuse large B cell lymphoma cells. J. Exp. Med..

[151] Hailfinger S., Lenz G., Ngo V., Posvitz-Fejfar A., Rebeaud F., Guzzardi M., Penas E.M., Dierlamm J., Chan W.C., Staudt L.M. (2009). Essential role of MALT1 protease activity in activated B cell-like diffuse large B-cell lymphoma. Proc. Natl. Acad. Sci. USA.

[152] Dai B., Grau M., Juilland M., Klener P., Horing E., Molinsky J., Schimmack G., Aukema S.M., Hoster E., Vogt N. (2017). B-cell receptor-driven MALT1 activity regulates MYC signaling in mantle cell lymphoma. Blood.

[153] Saba N.S., Wong D.H., Tanios G., Iyer J.R., Lobelle-Rich P., Dadashian E.L., Liu D., Fontan L., Flemington E.K., Nichols C.M. (2017). MALT1 Inhibition Is Efficacious in Both Naive and Ibrutinib-Resistant Chronic Lymphocytic Leukemia. Cancer Res..

[154] Fontan L., Yang C., Kabaleeswaran V., Volpon L., Osborne M.J., Beltran E., Garcia M., Cerchietti L., Shaknovich R., Yang S.N. (2012). MALT1 small molecule inhibitors specifically suppress ABC-DLBCL in vitro and in vivo. Cancer Cell.

[155] Nagel D., Spranger S., Vincendeau M., Grau M., Raffegerst S., Kloo B., Hlahla D., Neuenschwander M., Peter von Kries J., Hadian K. (2012). Pharmacologic inhibition of MALT1 protease by phenothiazines as a therapeutic approach for the treatment of aggressive ABC-DLBCL. Cancer Cell.

[156] Bardet M., Unterreiner A., Malinverni C., Lafossas F., Vedrine C., Boesch D., Kolb Y., Kaiser D., Gluck A., Schneider M.A. (2018). The T-cell fingerprint of MALT1 paracaspase revealed by selective inhibition. Immunol. Cell Biol..

[157] Wertz I.E., O’Rourke K.M., Zhou H., Eby M., Aravind L., Seshagiri S., Wu P., Wiesmann C., Baker R., Boone D.L. (2004). De-ubiquitination and ubiquitin ligase domains of A20 downregulate NF-κB signalling. Nature.

[158] Tavares R.M., Turer E.E., Liu C.L., Advincula R., Scapini P., Rhee L., Barrera J., Lowell C.A., Utz P.J., Malynn B.A. (2010). The ubiquitin modifying enzyme A20 restricts B cell survival and prevents autoimmunity. Immunity.

[159] Hymowitz S.G., Wertz I.E. (2010). A20: From ubiquitin editing to tumour suppression. Nat. Rev. Cancer.

[160] Compagno M., Lim W.K., Grunn A., Nandula S.V., Brahmachary M., Shen Q., Bertoni F., Ponzoni M., Scandurra M., Califano A. (2009). Mutations of multiple genes cause deregulation of NF-κB in diffuse large B-cell lymphoma. Nature.

[161] Honma K., Tsuzuki S., Nakagawa M., Tagawa H., Nakamura S., Morishima Y., Seto M. (2009). TNFAIP3/A20 functions as a novel tumor suppressor gene in several subtypes of non-Hodgkin lymphomas. Blood.

[162] Schmitz R., Hansmann M.L., Bohle V., Martin-Subero J.I., Hartmann S., Mechtersheimer G., Klapper W., Vater I., Giefing M., Gesk S. (2009). TNFAIP3 (A20) is a tumor suppressor gene in Hodgkin lymphoma and primary mediastinal B cell lymphoma. J. Exp. Med..

[163] Kato M., Sanada M., Kato I., Sato Y., Takita J., Takeuchi K., Niwa A., Chen Y., Nakazaki K., Nomoto J. (2009). Frequent inactivation of A20 in B-cell lymphomas. Nature.

[164] Chu Y., Vahl J.C., Kumar D., Heger K., Bertossi A., Wojtowicz E., Soberon V., Schenten D., Mack B., Reutelshofer M. (2011). B cells lacking the tumor suppressor TNFAIP3/A20 display impaired differentiation and hyperactivation and cause inflammation and autoimmunity in aged mice. Blood.

[165] Yang Y., Schmitz R., Mitala J., Whiting A., Xiao W., Ceribelli M., Wright G.W., Zhao H., Yang Y., Xu W. (2014). Essential role of the linear ubiquitin chain assembly complex in lymphoma revealed by rare germline polymorphisms. Cancer Discov..

[166] Dubois S.M., Alexia C., Wu Y., Leclair H.M., Leveau C., Schol E., Fest T., Tarte K., Chen Z.J., Gavard J. (2014). A catalytic-independent role for the LUBAC in NF-κB activation upon antigen receptor engagement and in lymphoma cells. Blood.

[167] Lin X., Mu Y., Cunningham E.T., Marcu K.B., Geleziunas R., Greene W.C. (1998). Molecular determinants of NF-κB-inducing kinase action. Mol. Cell Biol.

[168] Senftleben U., Cao Y., Xiao G., Greten F.R., Krahn G., Bonizzi G., Chen Y., Hu Y., Fong A., Sun S.C. (2001). Activation by IKKα of a second, evolutionary conserved, NF-κB signaling pathway. Science.

[169] Ranuncolo S.M., Pittaluga S., Evbuomwan M.O., Jaffe E.S., Lewis B.A. (2012). Hodgkin lymphoma requires stabilized NIK and constitutive RelB expression for survival. Blood.

[170] Otto C., Giefing M., Massow A., Vater I., Gesk S., Schlesner M., Richter J., Klapper W., Hansmann M.L., Siebert R. (2012). Genetic lesions of the TRAF3 and MAP3K14 genes in classical Hodgkin lymphoma. Br. J. Haematol..

[171] Liao G., Zhang M., Harhaj E.W., Sun S.C. (2004). Regulation of the NF-κB-inducing kinase by tumor necrosis factor receptor-associated factor 3-induced degradation. J. Biol. Chem..

[172] Zhang B., Calado D.P., Wang Z., Frohler S., Kochert K., Qian Y., Koralov S.B., Schmidt-Supprian M., Sasaki Y., Unitt C. (2015). An oncogenic role for alternative NF-κB signaling in DLBCL revealed upon deregulated BCL6 expression. Cell Rep..

[173] Hildebrand J.M., Luo Z., Manske M.K., Price-Troska T., Ziesmer S.C., Lin W., Hostager B.S., Slager S.L., Witzig T.E., Ansell S.M. (2010). A BAFF-R mutation associated with non-Hodgkin lymphoma alters TRAF recruitment and reveals new insights into BAFF-R signaling. J. Exp. Med..

[174] Guo X., Koff J.L., Moffitt A.B., Cinar M., Ramachandiran S., Chen Z., Switchenko J.M., Mosunjac M., Neill S.G., Mann K.P. (2017). Molecular impact of selective NFKB1 and NFKB2 signaling on DLBCL phenotype. Oncogene.

[175] Gruss H.J., Kadin M.E. (1996). Pathophysiology of Hodgkin’s disease: Functional and molecular aspects. Baillieres Clin. Haematol..

[176] Deacon E.M., Pallesen G., Niedobitek G., Crocker J., Brooks L., Rickinson A.B., Young L.S. (1993). Epstein-Barr virus and Hodgkin’s disease: Transcriptional analysis of virus latency in the malignant cells. J. Exp. Med..

[177] Kilger E., Kieser A., Baumann M., Hammerschmidt W. (1998). Epstein-Barr virus-mediated B-cell proliferation is dependent upon latent membrane protein 1, which simulates an activated CD40 receptor. EMBO J..

[178] Eliopoulos A.G., Caamano J.H., Flavell J., Reynolds G.M., Murray P.G., Poyet J.L., Young L.S. (2003). Epstein-Barr virus-encoded latent infection membrane protein 1 regulates the processing of p100 NF-κB2 to p52 via an IKKγ/NEMO-independent signalling pathway. Oncogene.

[179] Graham J.P., Arcipowski K.M., Bishop G.A. (2010). Differential B-lymphocyte regulation by CD40 and its viral mimic, latent membrane protein 1. Immunol. Rev..

[180] Luftig M., Prinarakis E., Yasui T., Tsichritzis T., Cahir-McFarland E., Inoue J., Nakano H., Mak T.W., Yeh W.C., Li X. (2003). Epstein-Barr virus latent membrane protein 1 activation of NF-κB through IRAK1 and TRAF6. Proc. Natl. Acad. Sci. USA.

[181] Xiao G., Cvijic M.E., Fong A., Harhaj E.W., Uhlik M.T., Waterfield M., Sun S.C. (2001). Retroviral oncoprotein Tax induces processing of NF-κB2/p100 in T cells: Evidence for the involvement of IKKα. EMBO J..

[182] Migliazza A., Lombardi L., Rocchi M., Trecca D., Chang C.C., Antonacci R., Fracchiolla N.S., Ciana P., Maiolo A.T., Neri A. (1994). Heterogeneous chromosomal aberrations generate 3′ truncations of the NFKB2/lyt-10 gene in lymphoid malignancies. Blood.

[183] Isogawa M., Higuchi M., Takahashi M., Oie M., Mori N., Tanaka Y., Aoyagi Y., Fujii M. (2008). Rearranged NF-κB2 gene in an adult T-cell leukemia cell line. Cancer Sci..

[184] Brummelkamp T.R., Nijman S.M., Dirac A.M., Bernards R. (2003). Loss of the cylindromatosis tumour suppressor inhibits apoptosis by activating NF-κB. Nature.

[185] Kovalenko A., Chable-Bessia C., Cantarella G., Israel A., Wallach D., Courtois G. (2003). The tumour suppressor CYLD negatively regulates NF-κB signalling by deubiquitination. Nature.

[186] Trompouki E., Hatzivassiliou E., Tsichritzis T., Farmer H., Ashworth A., Mosialos G. (2003). CYLD is a deubiquitinating enzyme that negatively regulates NF-κB activation by TNFR family members. Nature.

[187] Ramakrishnan P., Wang W., Wallach D. (2004). Receptor-specific signaling for both the alternative and the canonical NF-κB activation pathways by NF-κB-inducing kinase. Immunity.

[188] Demchenko Y.N., Brents L.A., Li Z., Bergsagel L.P., McGee L.R., Kuehl M.W. (2014). Novel inhibitors are cytotoxic for myeloma cells with NFkB inducing kinase-dependent activation of NFkB. Oncotarget.

[189] Castanedo G.M., Blaquiere N., Beresini M., Bravo B., Brightbill H., Chen J., Cui H.F., Eigenbrot C., Everett C., Feng J. (2017). Structure-Based Design of Tricyclic NF-κB Inducing Kinase (NIK) Inhibitors That Have High Selectivity over Phosphoinositide-3-kinase (PI3K). J. Med. Chem..

[190] Huxford T., Huang D.B., Malek S., Ghosh G. (1998). The crystal structure of the IκBα/NF-κB complex reveals mechanisms of NF-κB inactivation. Cell.

[191] Ito C.Y., Adey N., Bautch V.L., Baldwin A.S. (1995). Structure and evolution of the human IKBA gene. Genomics.

[192] Beg A.A., Sha W.C., Bronson R.T., Baltimore D. (1995). Constitutive NF-κB activation, enhanced granulopoiesis, and neonatal lethality in IκBα-deficient mice. Genes Dev..

[193] Cabannes E., Khan G., Aillet F., Jarrett R.F., Hay R.T. (1999). Mutations in the IkBa gene in Hodgkin’s disease suggest a tumour suppressor role for IκBα. Oncogene.

[194] Jungnickel B., Staratschek-Jox A., Brauninger A., Spieker T., Wolf J., Diehl V., Hansmann M.L., Rajewsky K., Kuppers R. (2000). Clonal deleterious mutations in the IκBα gene in the malignant cells in Hodgkin’s lymphoma. J. Exp. Med..

[195] Kanzler H., Kuppers R., Hansmann M.L., Rajewsky K. (1996). Hodgkin and Reed-Sternberg cells in Hodgkin’s disease represent the outgrowth of a dominant tumor clone derived from (crippled) germinal center B cells. J. Exp. Med..

[196] Krappmann D., Emmerich F., Kordes U., Scharschmidt E., Dorken B., Scheidereit C. (1999). Molecular mechanisms of constitutive NF-κB/Rel activation in Hodgkin/Reed-Sternberg cells. Oncogene.

[197] Staudt L.M. (2000). The molecular and cellular origins of Hodgkin’s disease. J. Exp. Med..

[198] Osborne J., Lake A., Alexander F.E., Taylor G.M., Jarrett R.F. (2005). Germline mutations and polymorphisms in the NFKBIA gene in Hodgkin lymphoma. Int. J. Cancer.

[199] Emmerich F., Meiser M., Hummel M., Demel G., Foss H.D., Jundt F., Mathas S., Krappmann D., Scheidereit C., Stein H. (1999). Overexpression of IκBα without inhibition of NF-κB activity and mutations in the IκBα gene in Reed-Sternberg cells. Blood.

[200] Liu X., Yu H., Yang W., Zhou X., Lu H., Shi D. (2010). Mutations of NFKBIA in biopsy specimens from Hodgkin lymphoma. Cancer Genet. Cytogenet..

[201] Lake A., Shield L.A., Cordano P., Chui D.T., Osborne J., Crae S., Wilson K.S., Tosi S., Knight S.J., Gesk S. (2009). Mutations of NFKBIA, encoding IκBα, are a recurrent finding in classical Hodgkin lymphoma but are not a unifying feature of non-EBV-associated cases. Int. J. Cancer.

[202] Thomas R.K., Wickenhauser C., Tawadros S., Diehl V., Kuppers R., Wolf J., Schmitz R. (2004). Mutational analysis of the IκBα gene in activated B cell-like diffuse large B-cell lymphoma. Br. J. Haematol..

[203] Takahashi H., Feuerhake F., Monti S., Kutok J.L., Aster J.C., Shipp M.A. (2006). Lack of IKBA coding region mutations in primary mediastinal large B-cell lymphoma and the host response subtype of diffuse large B-cell lymphoma. Blood.

[204] Johansson P., Klein-Hitpass L., Grabellus F., Arnold G., Klapper W., Pfortner R., Duhrsen U., Eckstein A., Durig J., Kuppers R. (2016). Recurrent mutations in NF-κB pathway components, KMT2D, and NOTCH1/2 in ocular adnexal MALT-type marginal zone lymphomas. Oncotarget.

[205] Emmerich F., Theurich S., Hummel M., Haeffker A., Vry M.S., Dohner K., Bommert K., Stein H., Dorken B. (2003). Inactivating IκBε mutations in Hodgkin/Reed-Sternberg cells. J. Pathol..

[206] Alves B.N., Tsui R., Almaden J., Shokhirev M.N., Davis-Turak J., Fujimoto J., Birnbaum H., Ponomarenko J., Hoffmann A. (2014). IκBε is a key regulator of B cell expansion by providing negative feedback on cRel and RelA in a stimulus-specific manner. J. Immunol..

[207] Mansouri L., Sutton L.A., Ljungstrom V., Bondza S., Arngarden L., Bhoi S., Larsson J., Cortese D., Kalushkova A., Plevova K. (2015). Functional loss of IκBε leads to NF-κB deregulation in aggressive chronic lymphocytic leukemia. J. Exp. Med..

[208] Mansouri L., Noerenberg D., Young E., Mylonas E., Abdulla M., Frick M., Asmar F., Ljungstrom V., Schneider M., Yoshida K. (2016). Frequent NFKBIE deletions are associated with poor outcome in primary mediastinal B-cell lymphoma. Blood.

[209] Whiteside S.T., Epinat J.C., Rice N.R., Israel A. (1997). IκBε, a novel member of the IκB family, controls RelA and cRel NF-κB activity. EMBO J..

[210] Lee S.H., Hannink M. (2002). Characterization of the nuclear import and export functions of IκB(ε). J. Biol. Chem..

[211] Memet S., Laouini D., Epinat J.C., Whiteside S.T., Goudeau B., Philpott D., Kayal S., Sansonetti P.J., Berche P., Kanellopoulos J. (1999). IκBε-deficient mice: Reduction of one T cell precursor subspecies and enhanced Ig isotype switching and cytokine synthesis. J. Immunol..

[212] Nencioni A., Grunebach F., Patrone F., Ballestrero A., Brossart P. (2007). Proteasome inhibitors: Antitumor effects and beyond. Leukemia.

[213] Jackson G., Einsele H., Moreau P., Miguel J.S. (2005). Bortezomib, a novel proteasome inhibitor, in the treatment of hematologic malignancies. Cancer Treat. Rev..

[214] Richardson P.G., Sonneveld P., Schuster M.W., Irwin D., Stadtmauer E.A., Facon T., Harousseau J.L., Ben-Yehuda D., Lonial S., Goldschmidt H. (2005). Bortezomib or high-dose dexamethasone for relapsed multiple myeloma. N. Engl. J. Med..

[215] Dunleavy K., Pittaluga S., Czuczman M.S., Dave S.S., Wright G., Grant N., Shovlin M., Jaffe E.S., Janik J.E., Staudt L.M. (2009). Differential efficacy of bortezomib plus chemotherapy within molecular subtypes of diffuse large B-cell lymphoma. Blood.

[216] Ohno H., Takimoto G., McKeithan T.W. (1990). The candidate proto-oncogene bcl-3 is related to genes implicated in cell lineage determination and cell cycle control. Cell.

[217] Kerr L.D., Duckett C.S., Wamsley P., Zhang Q., Chiao P., Nabel G., McKeithan T.W., Baeuerle P.A., Verma I.M. (1992). The proto-oncogene bcl-3 encodes an IκB protein. Genes Dev..

[218] Wulczyn F.G., Naumann M., Scheidereit C. (1992). Candidate proto-oncogene bcl-3 encodes a subunit-specific inhibitor of transcription factor NF-κB. Nature.

[219] Hatada E.N., Nieters A., Wulczyn F.G., Naumann M., Meyer R., Nucifora G., McKeithan T.W., Scheidereit C. (1992). The ankyrin repeat domains of the NF-κB precursor p105 and the protooncogene bcl-3 act as specific inhibitors of NF-κB DNA binding. Proc. Natl. Acad. Sci. USA.

[220] Bours V., Franzoso G., Azarenko V., Park S., Kanno T., Brown K., Siebenlist U. (1993). The oncoprotein Bcl-3 directly transactivates through kappa B motifs via association with DNA-binding p50B homodimers. Cell.

[221] Fujita T., Nolan G.P., Liou H.C., Scott M.L., Baltimore D. (1993). The candidate proto-oncogene bcl-3 encodes a transcriptional coactivator that activates through NF-κB p50 homodimers. Genes Dev..

[222] Carmody R.J., Ruan Q., Palmer S., Hilliard B., Chen Y.H. (2007). Negative regulation of toll-like receptor signaling by NF-κB p50 ubiquitination blockade. Science.

[223] Dechend R., Hirano F., Lehmann K., Heissmeyer V., Ansieau S., Wulczyn F.G., Scheidereit C., Leutz A. (1999). The Bcl-3 oncoprotein acts as a bridging factor between NF-κB/Rel and nuclear co-regulators. Oncogene.

[224] Viatour P., Dejardin E., Warnier M., Lair F., Claudio E., Bureau F., Marine J.C., Merville M.P., Maurer U., Green D. (2004). GSK3-mediated BCL-3 phosphorylation modulates its degradation and its oncogenicity. Mol. Cell.

[225] Na S.Y., Choi J.E., Kim H.J., Jhun B.H., Lee Y.C., Lee J.W. (1999). Bcl3, an IκB protein, stimulates activating protein-1 transactivation and cellular proliferation. J. Biol. Chem..

[226] Canoz O., Rassidakis G.Z., Admirand J.H., Medeiros L.J. (2004). Immunohistochemical detection of BCL-3 in lymphoid neoplasms: A survey of 353 cases. Mod. Pathol..

[227] McKeithan T.W., Takimoto G.S., Ohno H., Bjorling V.S., Morgan R., Hecht B.K., Dube I., Sandberg A.A., Rowley J.D. (1997). BCL3 rearrangements and t(14;19) in chronic lymphocytic leukemia and other B-cell malignancies: A molecular and cytogenetic study. Genes Chromosomes Cancer.

[228] Michaux L., Dierlamm J., Wlodarska I., Bours V., Van den Berghe H., Hagemeijer A. (1997). t(14;19)/BCL3 rearrangements in lymphoproliferative disorders: A review of 23 cases. Cancer Genet. Cytogenet..

[229] Martin-Subero J.I., Wlodarska I., Bastard C., Picquenot J.M., Hoppner J., Giefing M., Klapper W., Siebert R. (2006). Chromosomal rearrangements involving the BCL3 locus are recurrent in classical Hodgkin and peripheral T-cell lymphoma. Blood.

[230] Rassidakis G.Z., Oyarzo M.P., Medeiros L.J. (2003). BCL-3 overexpression in anaplastic lymphoma kinase-positive anaplastic large cell lymphoma. Blood.

[231] Nishikori M., Maesako Y., Ueda C., Kurata M., Uchiyama T., Ohno H. (2003). High-level expression of BCL3 differentiates t(2;5)(p23;q35)-positive anaplastic large cell lymphoma from Hodgkin disease. Blood.

[232] Ong S.T., Hackbarth M.L., Degenstein L.C., Baunoch D.A., Anastasi J., McKeithan T.W. (1998). Lymphadenopathy, splenomegaly, and altered immunoglobulin production in BCL3 transgenic mice. Oncogene.

[233] Westerheide S.D., Mayo M.W., Anest V., Hanson J.L., Baldwin A.S. (2001). The putative oncoprotein Bcl-3 induces cyclin D1 to stimulate G(1) transition. Mol. Cell. Biol..

[234] Mitchell T.C., Hildeman D., Kedl R.M., Teague T.K., Schaefer B.C., White J., Zhu Y., Kappler J., Marrack P. (2001). Immunological adjuvants promote activated T cell survival via induction of Bcl-3. Nat. Immunol..

[235] Kitamura H., Kanehira K., Okita K., Morimatsu M., Saito M. (2000). MAIL, a novel nuclear IκB protein that potentiates LPS-induced IL-6 production. FEBS Lett..

[236] Haruta H., Kato A., Todokoro K. (2001). Isolation of a novel interleukin-1-inducible nuclear protein bearing ankyrin-repeat motifs. J. Biol. Chem..

[237] Motoyama M., Yamazaki S., Eto-Kimura A., Takeshige K., Muta T. (2005). Positive and negative regulation of nuclear factor-kappaB-mediated transcription by IκB-ζ, an inducible nuclear protein. J. Biol. Chem..

[238] Tartey S., Matsushita K., Vandenbon A., Ori D., Imamura T., Mino T., Standley D.M., Hoffmann J.A., Reichhart J.M., Akira S. (2014). Akirin2 is critical for inducing inflammatory genes by bridging IκB-ζ and the SWI/SNF complex. EMBO J..

[239] Yamamoto M., Yamazaki S., Uematsu S., Sato S., Hemmi H., Hoshino K., Kaisho T., Kuwata H., Takeuchi O., Takeshige K. (2004). Regulation of Toll/IL-1-receptor-mediated gene expression by the inducible nuclear protein IκBζ. Nature.

[240] Okamoto K., Iwai Y., Oh-Hora M., Yamamoto M., Morio T., Aoki K., Ohya K., Jetten A.M., Akira S., Muta T. (2010). IκBζ regulates T(H)17 development by cooperating with ROR nuclear receptors. Nature.

[241] Chapuy B., Roemer M.G., Stewart C., Tan Y., Abo R.P., Zhang L., Dunford A.J., Meredith D.M., Thorner A.R., Jordanova E.S. (2016). Targetable genetic features of primary testicular and primary central nervous system lymphomas. Blood.

[242] Nogai H., Wenzel S.S., Hailfinger S., Grau M., Kaergel E., Seitz V., Wollert-Wulf B., Pfeifer M., Wolf A., Frick M. (2013). IκB-ζ controls the constitutive NF-κB target gene network and survival of ABC DLBCL. Blood.

[243] Kimura R., Senba M., Cutler S.J., Ralph S.J., Xiao G., Mori N. (2013). Human T cell leukemia virus type I tax-induced IκB-ζ modulates tax-dependent and tax-independent gene expression in T cells. Neoplasia.

[244] Ishikawa C., Senba M., Mori N. (2015). Induction of IκB-ζ by Epstein-Barr virus latent membrane protein-1 and CD30. Int. J. Oncol..

[245] Collins P.E., Kiely P.A., Carmody R.J. (2014). Inhibition of transcription by B cell Leukemia 3 (Bcl-3) protein requires interaction with nuclear factor kappaB (NF-κB) p50. J. Biol. Chem..

[246] Nakanishi C., Toi M. (2005). Nuclear factor-kappaB inhibitors as sensitizers to anticancer drugs. Nat. Rev. Cancer.

[247] Li Q., Van Antwerp D., Mercurio F., Lee K.F., Verma I.M. (1999). Severe liver degeneration in mice lacking the IκB kinase 2 gene. Science.

[248] Tanaka M., Fuentes M.E., Yamaguchi K., Durnin M.H., Dalrymple S.A., Hardy K.L., Goeddel D.V. (1999). Embryonic lethality, liver degeneration, and impaired NF-κB activation in IKK-β-deficient mice. Immunity.

[249] Rudolph D., Yeh W.C., Wakeham A., Rudolph B., Nallainathan D., Potter J., Elia A.J., Mak T.W. (2000). Severe liver degeneration and lack of NF-κB activation in NEMO/IKKγ-deficient mice. Genes Dev..

[250] Gamble C., McIntosh K., Scott R., Ho K.H., Plevin R., Paul A. (2012). Inhibitory kappa B Kinases as targets for pharmacological regulation. Br. J. Pharmacol..

[251] Wullaert A., Bonnet M.C., Pasparakis M. (2011). NF-κB in the regulation of epithelial homeostasis and inflammation. Cell Res..

[252] Greten F.R., Arkan M.C., Bollrath J., Hsu L.C., Goode J., Miething C., Goktuna S.I., Neuenhahn M., Fierer J., Paxian S. (2007). NF-κB is a negative regulator of IL-1β secretion as revealed by genetic and pharmacological inhibition of IKKβ. Cell.

[253] Honigberg L.A., Smith A.M., Sirisawad M., Verner E., Loury D., Chang B., Li S., Pan Z., Thamm D.H., Miller R.A. (2010). The Bruton tyrosine kinase inhibitor PCI-32765 blocks B-cell activation and is efficacious in models of autoimmune disease and B-cell malignancy. Proc. Natl. Acad. Sci. USA.

[254] Herman S.E., Gordon A.L., Hertlein E., Ramanunni A., Zhang X., Jaglowski S., Flynn J., Jones J., Blum K.A., Buggy J.J. (2011). Bruton tyrosine kinase represents a promising therapeutic target for treatment of chronic lymphocytic leukemia and is effectively targeted by PCI-32765. Blood.

[255] Friedberg J.W., Sharman J., Sweetenham J., Johnston P.B., Vose J.M., Lacasce A., Schaefer-Cutillo J., De Vos S., Sinha R., Leonard J.P. (2010). Inhibition of Syk with fostamatinib disodium has significant clinical activity in non-Hodgkin lymphoma and chronic lymphocytic leukemia. Blood.

[256] Naylor T.L., Tang H., Ratsch B.A., Enns A., Loo A., Chen L., Lenz P., Waters N.J., Schuler W., Dorken B. (2011). Protein kinase C inhibitor sotrastaurin selectively inhibits the growth of CD79 mutant diffuse large B-cell lymphomas. Cancer Res..

[257] Zhang S.Q., Smith S.M., Zhang S.Y., Lynn Wang Y. (2015). Mechanisms of ibrutinib resistance in chronic lymphocytic leukaemia and non-Hodgkin lymphoma. Br. J. Haematol..

[258] Nagel D., Bognar M., Eitelhuber A.C., Kutzner K., Vincendeau M., Krappmann D. (2015). Combinatorial BTK and MALT1 inhibition augments killing of CD79 mutant diffuse large B cell lymphoma. Oncotarget.

[259] Gardam S., Beyaert R. (2011). The kinase NIK as a therapeutic target in multiple myeloma. Expert Opin. Ther. Targets.

